# A Liquid Chromatography-Tandem Mass Spectrometry Method for the Quantification of Cystic Fibrosis Drugs (Caftors) in Plasma and Its Application for Therapeutic Monitoring

**DOI:** 10.3390/molecules30091866

**Published:** 2025-04-22

**Authors:** Valentina D’Atri, Fabrizio Corrado, François Versace, Susana Alves Saldanha, Thomas Mercier, Monia Guidi, Paul Thoueille, Sylvain Blanchon, Angela Koutsokera, Michael Vogeser, Catia Marzolini, François Girardin, Georgia Mitropoulou, Zisis Balmpouzis, Isabelle Rochat, Alain Sauty, Laurent Arthur Decosterd, Eva Choong

**Affiliations:** 1 Service and Laboratory of Clinical Pharmacology, Department of Laboratory Medicine and Pathology, Lausanne University Hospital and University of Lausanne, 1011 Lausanne, Switzerland; 2 Center for Research and Innovation in Clinical Pharmaceutical Sciences, University Hospital and University of Lausanne, 1011 Lausanne, Switzerland; 3 Pediatric Pneumology and Cystic Fibrosis Unit, Division of Pediatrics, Department Woman-Mother-Child, Lausanne University Hospital and University of Lausanne, 1011 Lausanne, Switzerland; 4 Adult Cystic Fibrosis and CFTR-Related Disorders Center, Division of Pulmonology, Lausanne University Hospital and University of Lausanne, 1011 Lausanne, Switzerland; 5 Institute of Laboratory Medicine, LMU University Hospital, 81377 Munich, Germany; 6 Division of Pulmonology, Department of Medicine, Neuchâtel Hospital Network, 2000 Neuchâtel, Switzerland

**Keywords:** cystic fibrosis, CFTR modulators, therapeutic drug monitoring, LC-MS/MS, pharmacokinetics, precision medicine, method development, quantification, validation

## Abstract

Cystic fibrosis (CF) is a life-threatening disorder caused by mutations in the CFTR gene, leading to defective chloride ion transport and thickened mucus in the respiratory and gastrointestinal systems. CFTR modulators, including ivacaftor, lumacaftor, tezacaftor, and elexacaftor, have improved patient outcomes, but interindividual pharmacokinetic variability and potential drug–drug interactions require therapeutic drug monitoring (TDM) for optimal efficacy and safety. In this context, a liquid chromatography–tandem mass spectrometry (LC-MS/MS) method has been developed and validated for the simultaneous quantification of CFTR modulators and their major active metabolites in human plasma to support pharmacokinetic studies and routine TDM. The multiplex LC-MS/MS assay was established using plasma protein precipitation, followed by chromatographic separation on an Xselect HSS T3 (Waters^®^) column and positive electrospray ionization mode detection. The method was validated based on FDA and EMA guidelines for specificity, linearity, accuracy (89.8–107.8%), repeatability (1.1–8.1%), intermediate fidelity (1.3–10.9%), matrix effects, and stability, demonstrating a robust performance with excellent precision and accuracy. International interlaboratory comparisons confirmed the reliability of the assay. The developed method can be applied for the clinical monitoring of caftors’ plasma concentrations and preliminary data suggest that it can also be applied to alternative matrices, such as breast milk. This method will serve to characterize caftors’ pharmacokinetic variability and monitor drug–drug interactions to further refine personalized dosing strategies and enhance precision medicine treatments for patients with CF.

## 1. Introduction

Cystic fibrosis (CF) is a life-threatening genetic disorder caused by mutations in the cystic fibrosis transmembrane conductance regulator (CFTR) gene, leading to dysfunctional chloride transport and thickened mucus secretions in multiple organ systems, particularly the lungs and pancreas. The introduction of CFTR modulators—currently, the four marketed are ivacaftor, lumacaftor, tezacaftor, and elexacaftor—in the clinic has revolutionized the life of people with CF (pwCF) by improving lung function and reducing exacerbations of the disease in patients with CF [1]. These drugs, collectively referred to as “caftors”, work by enhancing CFTR function, either by correcting protein folding (currently, three marketed “correctors”: lumacaftor (LUM), tezacaftor (TEZ), and elexacaftor (ELX), or potentiating its activity (currently one marketed “potentiator”: ivacaftor (IVA)), used alone or in combination with correctors, so far, Orkambi^®^ (IVA + LUM combination therapy) or Trikafta^®^ (IVA + TEZ + ELX combination therapy) [1,2]. Caftors are subject to important interindividual pharmacokinetic (PK) variability. These drugs are metabolized by CYP3A4 and therefore are also prone to drug–drug interactions, for instance, inducers can reduce their exposure, which may lead to a loss of clinical effect, while inhibitors can increase their exposure and the related risk of adverse effects. For instance, the IVA label recommends monitoring in case of coadministration with moderate inducers, while it indicates to reduce the IVA dose from 150 mg twice daily to 150 mg twice weekly with the concurrent use of strong CYP3A4 inhibitors [3]. Given the important interindividual pharmacokinetic variability, routine therapeutic drug monitoring (TDM) may be useful to monitor drug–drug interactions and tailor the dose adjustments of caftors [2,4]. It is indeed increasingly acknowledged that the full clinical benefits of these revolutionary caftors may be attained through their careful therapeutic drug monitoring (TDM) in patients, as is the case for other drug classes [5,6].

Liquid chromatography–tandem mass spectrometry (LC-MS/MS) has emerged as a gold-standard technique for the precise and sensitive quantification of small-molecule drugs in biological matrices.

Recent studies have focused on analytical methodologies for quantifying CFTR modulators. A high-resolution mass spectrometry (LC-HRMS) method for the quantification of IVA, LUM, TEZ, and ELX in human plasma and in breast milk was recently developed [7]. However, this method did not consider the metabolites ivacaftor-M1 (IVA-M1) and elexacaftor-M23 (ELX-M23), which represent an important part of the active species circulating in plasma and contribute to the overall activity, and thus deserve to be also monitored [2]. Another LC-MS/MS method has been proposed for the rapid quantification of IVA, LUM, ELX, TEZ, ivacaftor-M6, and IVA-M1 in plasma, yet without including tezacaftor-M1 (TEZ-M1) and ELX-M23 [8]. Similarly, an LC-MS/MS method for dried blood spot analysis was developed to quantify IVA, ivacaftor-M6, ELX, ELX-M23, TEZ, and TEZ-M1, yet omitting LUM [9]. Özcan et al. reported an LC-MS approach for LUM and IVA, primarily focusing on identifying five novel degradation products rather than active metabolites [10]. Other studies have also been limited in scope, with one focusing on ELX, TEZ, and IVA in human plasma [11], and in human plasma and cellular lysate [12]. Finally, an LC-MS/MS method described the dosages of IVA, IVA-M1, and the rather inactive ivacaftor-M6 (activity of 1/50th relative to IVA [3]), along with LUM and TEZ, but omitted ELX and ELX-M23 [13].

In this report, we describe the development and validation of an LC-MS/MS method for the simultaneous quantification of IVA, LUM, TEZ, and ELX, along with their major active metabolites: IVA-M1 (potency of 1/6th of that of IVA), TEZ-M1 (similar potency to that of TEZ), and ELX-M23 (exhibits similar potency to ELX) [14]. This approach incorporates stable isotope-labeled internal standards for all the parent drugs, enhancing the analytical accuracy and precision and supporting a reliable pharmacokinetic (PK) assessment. The method was developed in accordance with the regulatory guidelines for bioanalytical method validation to ensure its suitability for clinical application. Furthermore, in the absence of an established proficiency program, inter-laboratory comparisons (ILCs) were conducted with two European laboratories to evaluate the method’s performance and accuracy.

The LC-MS/MS method has a high sensitivity (demonstrated by its low limit of quantification, LLOQ) and a high specificity, particularly through the use of stable isotope-labeled internal standards, comparable in these terms with some other recent methods. However, the distinctive advantage of our method lies in its global and complete analysis of all the analytes of interest, thus overcoming the limitations observed in other methodologies that do not cover certain analytes, and offering a robust and thorough validation for all the analytes, including extensive stability and interference tests.

Furthermore, this method has been applied in a real-world clinical setting to evaluate drug exposure in patients with CF receiving CFTR modulator therapy, which constitutes a first step toward the emerging field of the precision therapy of CFTR disorders.

The proposed method constitutes an important analytical tool for facilitating PK studies and supporting personalized medicine approaches in CF care.

## 2. Results and Discussion

### 2.1. Analytical Method Development

Several LC and MS parameters were optimized. The LC parameters that were investigated included mobile phase composition (using either 0.1% FA or 0.2% FA in water), different elution programs (by gradually increasing the initial percentage of mobile phase B to 5, 15, 25, 35, 45, or 60%), and the modulation of the elution curve. Regarding the column length, two options were examined to obtain the best resolution: 75 mm (Waters C18 Xselect^®^ HSS T3, 3.5 µm, 2.1 × 75 mm) and 150 mm (Waters C18 Xselect^®^ HSS T3, 2.5 µm, 2.1 × 150 mm). In terms of resolution and selectivity, the 75 mm column provided faster analysis times while maintaining an adequate resolution for closely eluting peaks (LUM and IVA) and comparable selectivity once the chromatographic conditions were optimized.

To optimize the gradient, a sample containing all the analytes of interest in MeOH was used. The progressive increase in the initial ACN gradient between each test did not affect the quality of separation, with negligible selectivity variations. In this context, an initial gradient condition of 50% B ACN was determined to be optimal for achieving a sufficiently short analysis time while remaining suitable for the column’s dead volume.

The evaluation of mobile phase A, consisting of either 0.1% FA or 0.2% FA in water, is reported in Figure S1. Although the retention times were similar for both the mobile phase A compositions, 0.2% FA in water provided a better ionization of the ions with slightly higher MS sensitivities, and it was therefore retained as mobile phase A composition in the final chromatographic program.

Optimized MS/MS parameters are reported in Table 1. All the selected transitions proved to be precise and reproducible for the analysis of caftors and their metabolites. To perform this optimization, each analyte was individually spiked at 1 µg/mL in MeOH.

However, the optimization of the vaporization temperature was necessary to increase the sensitivity of the analysis for certain compounds, such as the metabolites. The optimization step involved successively injecting a solution containing all the molecules of interest while varying the vaporization temperature (40, 100, 150, 200, 250, and 300 °C). The peak areas obtained during the variation of the vaporization temperature are reported in Figure S2. As shown, the peak areas generally increased gradually with the increase in the vaporization temperature, except for IVA, which had a maximum at 150 °C, after which a slight decrease was observed. The low sensitivity that was generally observed for TEZ-M1 and ELX-M23 has dictated the optimized conditions that allowed their highest signals. Considering the sensitivity of all the analytes, the vaporization temperature of 300 °C was finally selected as the best compromise.

The sample precipitation procedure was also subjected to optimization. A comparison of two precipitation solvents, namely MeOH and ACN, based on the visual examination of the obtained extracts, and their impact on the peak’s shapes and the intensities of the different analytes, is reported in Figure S3. As shown, a slight difference between the two tested solvents was observed, with the peak areas of the analytes obtained using MeOH generally lower than those obtained with ACN. To account for the low analytical sensitivity observed for the metabolites, ACN was finally chosen. 

**Figure 1:**
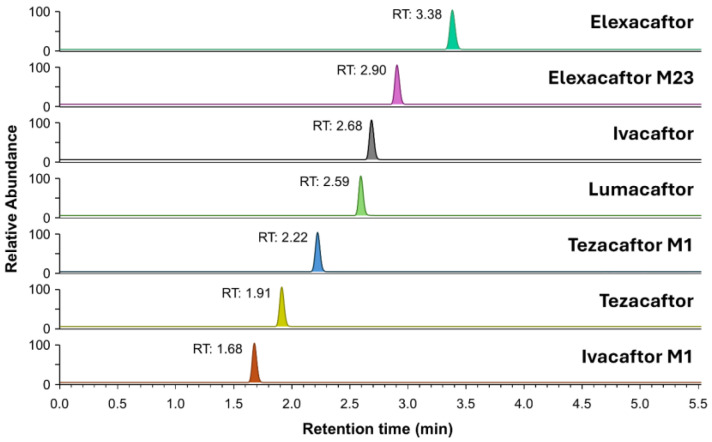
Multiplex plasma analysis using UHPLC-MS/MS of the four caftors and active metabolites. For the sake of readability, the corresponding internal standards are not shown. Chromatographic profile of a CAL6 plasma quality control sample (IVA 5 µg/mL, LUM 40 µg/mL, TEZ 10 µg/mL, ELX 15 µg/mL, IVA-M1 5 µg/mL, TEZ-M1 15 µg/mL, and ELX-M23 15 µg/mL).

The chromatographic profiles of the four caftors and active metabolites obtained after LC-MS/MS optimization are shown in Figure 1.

### 2.2. Validation of the Method

#### 2.2.1. Selectivity, Specificity, Crosstalk, and Carryover

The selectivity of the method was demonstrated by the absence of peaks (or values below the limit of acceptance) in several plasma samples from subjects not receiving caftors (i.e., blank plasma) (Table S1). Additionally, no crosstalk was observed between different analytes or between analytes and their respective internal standards (ISs). Figure S4 shows that LUM glucuronide is detected at a different retention time than the parent drug, which is necessary to prevent the overestimation in the quantification of LUM due to the in-source metabolite dissociation to the parent drug. In addition, an interference test with concomitant medications that are expected to be used in the treatment of patients with CF was also performed. Figure S6 highlights the chromatographic profile of the caftors’ QC3 plasma quality control sample in comparison with the known, high concentration of the potential interfering substances that are relevant to the patient population, namely ibuprofen, cortisone, several antibiotics (colistin, imipenem, cefepime, meropenem, cefazolin, piperacillin, amoxicillin, ceftazidime, ceftriaxone, daptomycin, ertapenem, flucloxacillin, and rifampicin), and antifungal drugs (fluconazole, voriconazole, posaconazole, OH-itraconazole, isavuconazole, and itraconazole). No interferences were detected.

Carryover was prevented by optimizing the liquid chromatography gradient program and implementing a double needle rinse step (ACN/MeOH/H_2_O 4:4:2). Additionally, a 98% B column washing step ensured the removal of residual contaminants, resulting in carryover levels below 15% relative to the area of the lowest calibrator.

#### 2.2.2. Limit of Detection and Linearity

The evaluation of the LODs was satisfactory, as it did not exceed a signal-to-noise ratio of 3:1. For ELX and TEZ-M1, a 10-fold sample dilution of the lowest calibration sample produced a signal that was nine times greater than the background noise. Further dilution would not be clinically relevant, as this 10-fold dilution corresponds already to a plasma concentration of 15 ng/mL for both TEZ-M1 and ELX, which are much lower than those encountered in patient samples.

The linearity observed during validation is considered satisfactory, with slopes ranging between 0.94 and 1.04. Additionally, all the determination coefficients (R^2^) obtained are greater than 0.99 (Table S2), demonstrating that the method reliably predicts the analyte concentrations across the tested range.

#### 2.2.3. Trueness and Precision

The accuracy and precision calculated from the analysis of QCs during the three validation days are within the defined range of ±15% of the target value for the seven analytes (Table 2).

**Figure 2:**
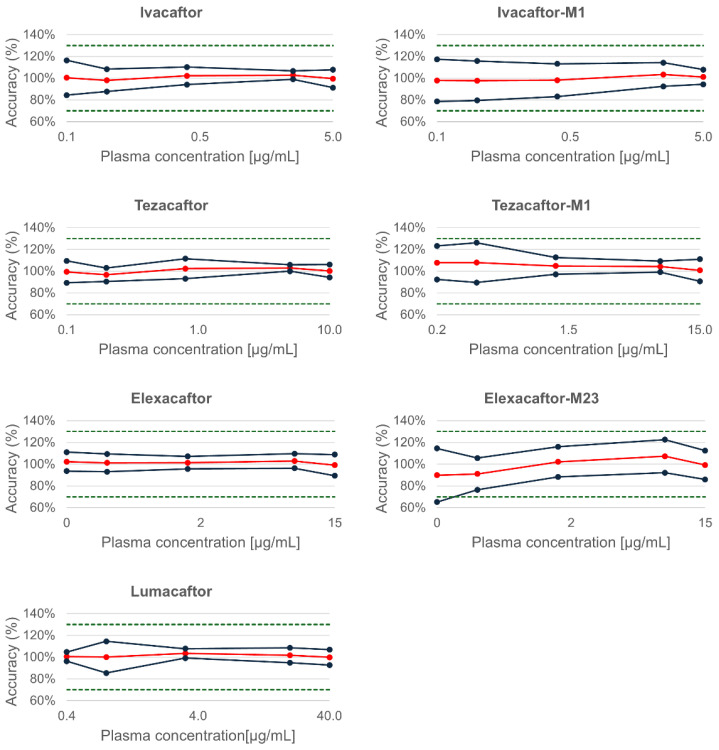
Accuracy profiles of the analytes and their metabolites on three sets of analyses with a tolerance interval of 30% and a risk α at 5%. Mean bias obtained over 3 days for each concentration of QC (five levels: L1, L2, M1, M2, H) are reported in red. Upper and lower β-expectation tolerance intervals (β = 95%, black lines) and acceptance limits (±30%, green dotted lines) are also presented.

The accuracy profiles (Figure 2) obtained for the analytes IVA, LUM, TEZ, ELX, and the metabolites IVA-M1 and TEZ-M1 were within the ±30% acceptance limits defined by the tolerance interval for biological samples. In contrast, a slightly higher bias was observed at the lowest quality control (QC) level (0.15 µg/mL) for ELX-M23. As a result, the lower limit of quantification (LLOQ) for ELX-M23 was conservatively established at 0.3 µg/mL. For all the other analytes, the LLOQ and upper limit of quantification (ULOQ) correspond to the lowest and highest validated concentrations, respectively, as reported in Table 3. This observation highlights a potential analytical limitation, which may be attributable to the absence of a compound-specific stable isotope-labeled internal standard for ELX-M23—currently not commercially available—potentially affecting the quantification accuracy at low concentrations.

#### 2.2.4. Evaluation of the Matrix Effect

**Figure 3:**
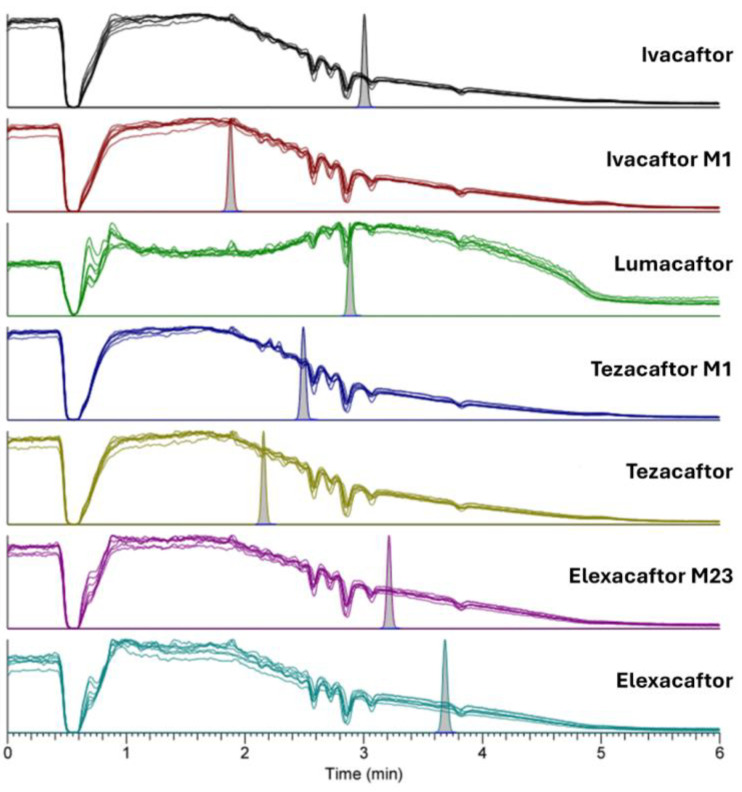
Qualitative evaluation of the matrix effect. Chromatograms of six different blank plasma extracts with post-column infusion of a CAL6 sample of each analyte (IVA 5 µg/mL, LUM 40 µg/mL, TEZ 10 µg/mL, ELX 15 µg/mL, IVA-M1 5 µg/mL, TEZ-M1 15 µg/mL, and ELX-M23 15 µg/mL).

During the qualitative test (Figure 3), potential signal suppression at the retention times of the analytes can be highlighted by overlaying the chromatograms obtained from analyzing the six blank plasmas, using the Matuszewski approach [15]. A slight indication of signal suppression near the retention times for certain analytes, such as IVA, LUM, or TEZ-M1, was observed. Nevertheless, the systematic use of stable isotopically labeled internal standards corrected for this matrix effect. The observed signal decrease between 2.5 and 3.2 min likely corresponds to the elution of phospholipids as they cross the MS/MS detector. In contrast, the complete signal suppression at 0.5 min is due to the column’s dead volume, likely caused by the presence of highly polar compounds that were not retained on the column.

#### 2.2.5. Stability Studies

**Figure 4:**
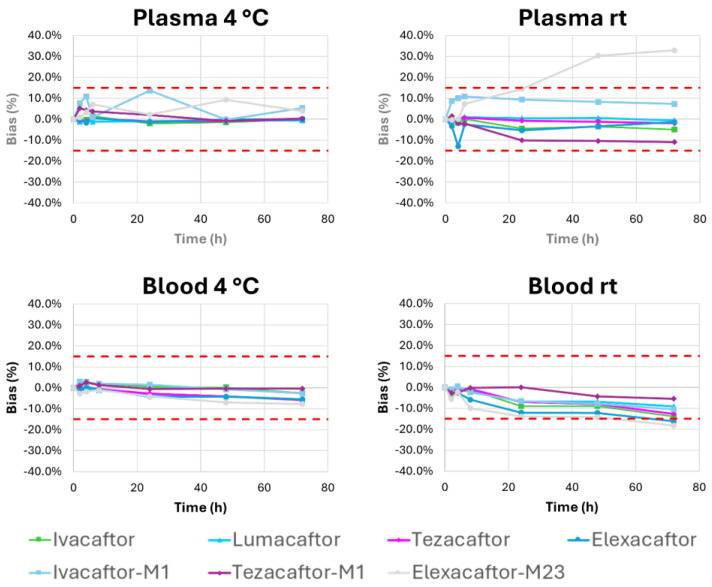
Short-term stability testing of all the analytes in plasma and whole blood. Quantification performed on three QC samples (low, medium, and high concentrations) over time (at 0, 2, 4, 6, 24, 48, and 72 h) at room temperature (rt) and at +4 °C. The mean deviation of the measured values from the nominal concentration at T0 for the three QC samples is shown. Upper and lower acceptance limits (±15%, red dotted lines) are also presented.

The deviations measured for the stability profiles of the parent molecules are less than 15%, indicating that the molecules IVA, LUM, TEZ, and ELX remain stable in plasma for 72 h, both at room temperature and at 4 °C (Figure 4). In contrast, the metabolites IVA-M1, TEZ-M1, and ELX-M23 remain stable in plasma for 72 h when stored at 4 °C but exhibit variable stability profiles at room temperature. The stability test results suggest that the blood samples should be centrifuged immediately after collection to allow the plasma to be sent within 72 h to the laboratory at room temperature for analysis (Figure 4).

**Figure 5:**
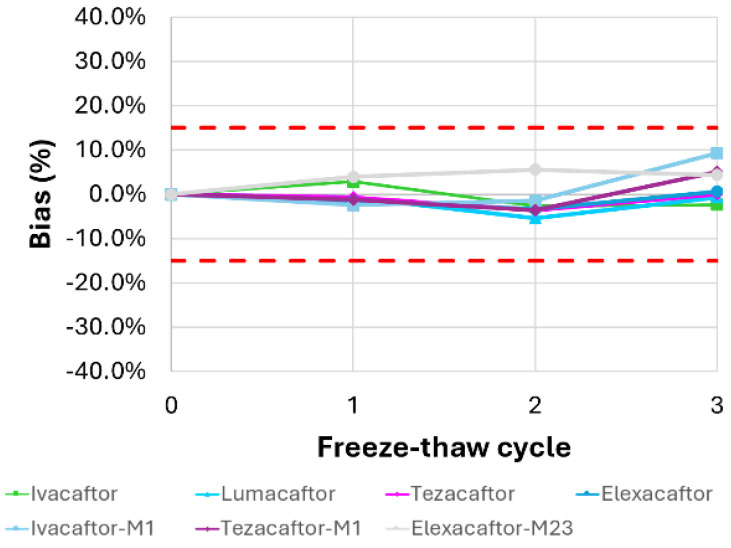
Freeze-thaw stability testing of all the analytes in plasma. Quantification was performed on three QC samples (low, medium, and high concentrations) over three freezing/thawing cycles. The mean deviation of the measured values from the nominal concentration at T0 for the three QC samples is shown. Upper and lower acceptance limits (±15%, red dotted lines) are also presented.

The analysis of samples in vials stored in the autosampler at 4 °C for 24 h demonstrated good stability for all analytes, remaining below the ±15% limit (Table S4). Similarly, data obtained after three freeze-thaw cycles reveal a mean deviation of ≤12.2% compared with the reference, demonstrating the samples’ ability to remain stable during the cycles (Figure 5).

Data reported for integrity to dilution (Table S5) showed that for all the dilutions using blank plasma, the ratios obtained for all compounds are within the ±15% acceptance range, demonstrating the robustness of the method for plasma dilutions.

### 2.3. Inter-Laboratory Comparisons

**Figure 6:**
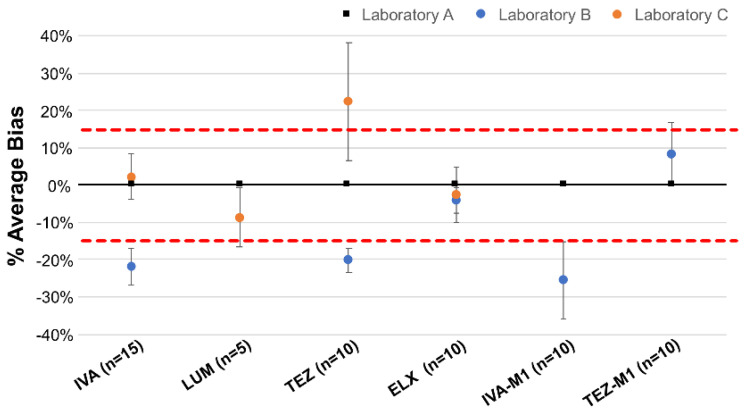
Inter-laboratory comparison. Results of the plasma concentrations obtained from samples analyzed simultaneously in our laboratory (Laboratory A) and two European laboratories (namely, Laboratory B or C).

The satisfactory performance of our laboratory in the inter-laboratory comparison (ILC) confirmed the accuracy of the analytical method. The exchange of drug samples among the collaborating laboratories demonstrated a high consistency in the measured concentrations, with minimal variation between the results (Figure 6). The fair agreement across laboratories validated the reliability of the developed analytical method, confirming its suitability for routine drug quantification. The scope and the strength of such an ILC program may certainly be improved if a growing number of laboratories performing TDM for caftors would be interested in participating (please contact the senior author for further information).

### 2.4. Clinical Application: Exploratory Analyses

**Figure 7:**
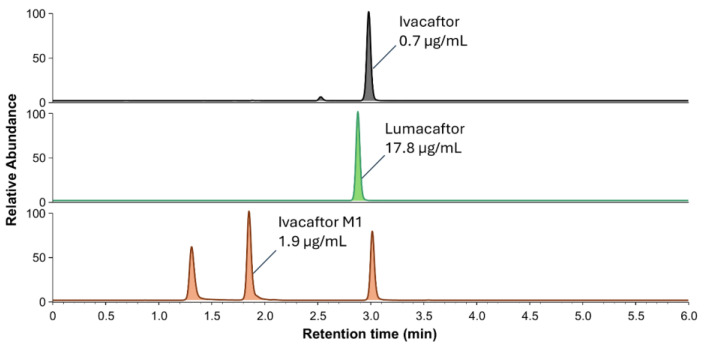
Chromatogram of a patient receiving IVA + LUM dual therapy, collected 3 h after dosing. The plasma concentrations of IVA, LUM, and IVA-M1 were 0.7, 17.8, and 1.9 µg/mL, respectively. The measured concentrations in this patient are in the range of expected concentrations based on available pharmacokinetic data [2]. Corresponding internal standards are not shown.

**Figure 8:**
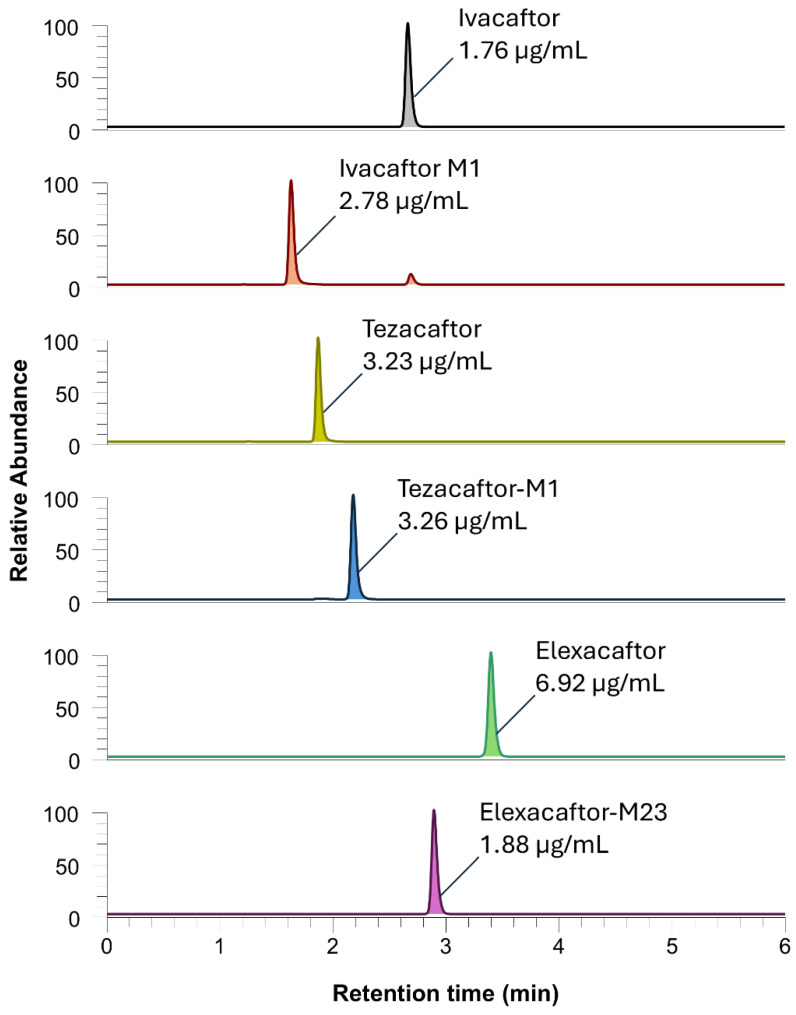
Chromatogram of a patient receiving IVA + TEZ + ELX triple therapy, collected 20 h after dosing. The plasma concentrations of IVA, IVA-M1, TEZ, TEZ-M1, ELX, and ELX-M23 were 1.76, 2.80, 3.39, 3.26, 6.92, and 1.88 µg/mL, respectively. The measured concentrations in this patient are higher than what has been previously reported [2]. Corresponding internal standards are not shown.

Two examples of the chromatographic profiles of patient samples are reported in Figure 7 and Figure 8. The first chromatogram (Figure 7) shows the signal peaks and concentrations obtained from a sample from a patient treated with IVA + LUM dual therapy. The sample was taken from the patient 3 h after the last dose of medication. The observed signals demonstrate the presence of the compounds of interest, including the active metabolite. The second series of chromatograms reported in Figure 8 represents the multiplex analysis of a plasma sample from a patient treated with IVA + TEZ + ELX triple therapy, 20 h after the drug intake. A quantification test was also performed on a breast milk sample from a consenting breast-feeding patient (Figure S5) treated with IVA + TEZ + ELX triple therapy, 3 h after the medication intake. A matrix-matched calibration with human milk was used for the quantification and QC in this matrix, and these were within the acceptance limit (i.e., bias < 15%). The reported examples demonstrate the specificity of the analytical method by highlighting the expected parent drugs and their respective metabolites. However, it should be noted that further validation is needed for the application of this method to breast milk. As the matrix is different from those originally tested, additional validation studies should be conducted to ensure the method’s accuracy and reliability for breast milk analysis.

Limited data exist regarding the plasma concentrations of caftors in pediatric populations, despite their increasing utilization in this patient group.

A phase II trial involving children aged 12–24 months reported IVA mean trough concentrations of ≥440 ng/mL, consistent with the previous findings in older children (2–5 years) and adults [16]. In a recent pharmacokinetic modeling study, Vonk et al. (2025) calculated the average concentrations (Cavg) of ELX, TEZ, and IVA in pediatric patients aged 6 years and older [17], which consistently exceeded the established effective concentration EC50 values (ELX: 500 ng/mL, TEZ: 400 ng/mL, IVA: 40 ng/mL) [18].

The lower limits of quantification (LLOQs) established in our validated analytical method included these observed plasma levels and effective cutoffs (i.e., LLOQ set at 50 ng/mL for IVA and IVA-M1, 400 ng/mL for LUM, 100 ng/mL for TEZ, and 150 ng/mL for ELX, TEZ-M1, and ELX-M23).

Moreover, the minimal required sample volume of 50 µL in our method is compatible with micro-sampling devices, such as Microvette (yielding approximately 100 µL plasma from 250 µL whole blood), or precision volumetric approaches like volumetric absorptive microsampling (VAMS, up to 120 µL whole blood) or other emerging devices [19].

Consequently, our method also shows potential applicability in pharmacokinetic monitoring in pediatric populations.

Data to date have shown that caftors are characterized by a large interindividual pharmacokinetic variability. Furthermore, phase II dose escalation studies have demonstrated a trend between increased clinical response and higher doses [2]. Altogether, the available data suggest that caftors are good candidates for TDM. However, future randomized controlled trials will need to establish the target concentrations for optimal therapeutic effect by comparing the clinical outcomes of patients with a TDM-guided dose adjustment with those receiving the standard recommended dose.

### 2.5. From Validation to Clinical Implementation

The bioanalytical method has been validated following ICH M10 guidelines [16] and demonstrates real-world application; however, more steps are required for its clinical implementation. The execution of one interlaboratory comparison with the two additional laboratories may be insufficient for widespread clinical laboratory adoption, particularly when pursuing compliance with accreditation standards for medical laboratories, including ISO 15189. Regulatory authorities and accreditation bodies typically require a robust demonstration of reproducibility and accuracy, which are conventionally achieved through participation in external quality assessment schemes (i.e., proficiency testing programs). This program would provide additional evidence supporting the method reliability; however, such proficiency testing protocols are currently unavailable for this specific novel therapeutic class. Therefore, establishing multiple independent, formally contracted interlaboratory comparisons conducted at regular intervals throughout the year constitutes an essential prerequisite for ensuring methodological accuracy and regulatory confidence.

Moreover, systematic documentation of comprehensive long-term stability studies for calibrators and all reagents should be prioritized, as these experimental protocols extend beyond the standard validation parameters. The current absence of certified reference materials necessitates attention to ensure metrological traceability; at a minimum, the utilization of reagents from different batches and/or suppliers would address the formal accreditation requirements.

The resolution of these methodological limitations would significantly enhance the analytical procedure’s suitability for broader clinical implementation and regulatory compliance. Such improvements represent a critical step toward fulfilling the fundamental requirements that are necessary to petition our national health authorities for reimbursement approval, ultimately enabling widespread clinical utilization and benefiting patient care.

## 3. Materials and Methods

### 3.1. Chemical and Reagents

The compounds analyzed in this study included IVA, LUM, TEZ, ELX, and their metabolites hydroxymethyl ivacaftor (i.e., IVA-M1), TEZ-M1, and ELX-M23. IVA (cat n° C48883), LUM (cat n° C49431), TEZ (cat n° C48881), ELX (cat n° C48878), and the isotope-labelled reference materials tezacaftor-D4 (TEZ-D4, cat n° C48882) and elexacaftor-D3 (ELX-D3, cat n° C48880) were obtained from Chemie Brunschwig AG (Basel, Switzerland). IVA-M1 (cat. n° CS-O-11850), TEZ-M1 (cat. n° CS-O-35941), ELX-M23 (cat. n° CS-O-35456), and the isotope-labelled reference materials ivacaftors-D4 (IVA-D4, cat. n° CS-O-01219), and lumacaftor-D4 (LUM-D4, cat. n° CS-P-08060) were obtained from Clearsynth (Brampton, ON, Canada). Voriconazole-D3 (VOR-D3, cat. n° TRC-V760002), found to be a suitable IS for IVA-M1, was purchased from LGC standards (Teddington, UK). Acetonitrile (ACN), methanol (MeOH), formic acid (FA), and dimethyl sulfoxide (DMSO) were of analytical grade (≥98%) and purchased from Merck (Darmstadt, Germany). Ultrapure water was supplied by a Milli-Q IQ 7000 purification system (Merck Millipore, Burlington, VT, USA).

### 3.2. Stock Solutions, Calibration, and Validation Standards Preparation

Stock solutions of the internal standards (ISs) IVA-D4 (5 mg/mL), LUM-D4 (2 mg/mL), TEZ-D4 (1 mg/mL), ELX-D3 (1 mg/mL), and VOR-D3 (5 mg/mL) were prepared by dilution in acetonitrile (ACN) to achieve final concentrations of 0.2 μg/mL, 1 μg/mL, 0.4 μg/mL, 0.4 μg/mL, and 0.5 mg/mL, respectively. The mix of IS was used to normalize the parent drug and the corresponding metabolite, except for IVA-M1, for which the optimal IS was voriconazole-D3 (Table 1).

A stock solution in DMSO was prepared for each analyte: IVA and LUM were solubilized to 5 mg/mL, while TEZ and ELX were solubilized to 2 mg/mL and 1 mg/mL, respectively. These stock solutions were subsequently stored at −80 °C. Two independent working solutions (WSs) were prepared by appropriately diluting the stock solutions in a mixture of H_2_O:MeOH (1:1). These were further diluted twenty-fold in plasma to produce the various calibrators (CALs) and quality controls (QCs) at the final concentrations specified in Table 3. Notably, the calibration concentration ranges were based on FDA data retrieved from the caftors’ drug registration files and from the comprehensive literature review of available clinical PK studies and encompass the plasma concentrations typically observed in patients [2,4,12,13,20,21,22].

### 3.3. Plasma Sample Extraction Procedure

Citrate plasma was donated from patients with Vaquez disease (polycythemia vera) undergoing their regular phlebotomy at the clinic “Unisanté”, University of Lausanne, Switzerland. This plasma, collected according to the institutional ethical standards, was used as “blank plasma” to generate the CAL and QC samples.

In 1.5-mL Eppendorf^®^ tubes, 50 µL of plasma sample (calibration, validation standards, quality control (QC), or patient samples) were precipitated with 150 µL of ACN containing the mix of IS (i.e., protein precipitation solvent). The mixture was vortexed and centrifugated at min 18,000× *g* (14,000 rpm) for 10 min at 4 °C using a Mikro 220R centrifuge from Hettich (Bäch, Switzerland) to achieve plasma protein precipitation. Subsequently, 150 µL of the supernatant were transferred to glass vials with inserts and mixed with 150 µL of MilliQ water before being vortexed again.

### 3.4. Instrumentation and Experimental Conditions

The analyses were performed on a LC-MS/MS system from ThermoFisher Scientific (San Jose, CA, USA), consisting of a Vanquish ultra-high-performance liquid chromatography (UHPLC) equipped with a 2-channel binary high-pressure gradient pump and a flow-through needle auto-sampler maintained at 4 °C. The chromatographic system was coupled with a TSQ Quantis triple quadrupole mass spectrometer equipped with an electrospray ionization (ESI) interface. The MS analyses were performed in ESI positive mode applying static spray voltage set to 3.45 kV. Of note, both positive and negative ESI modes were initially considered, but the negative mode was found to be less sensitive. The optimized ESI source parameter settings involved ion transfer tube and vaporizer temperatures of 350 °C and 300 °C, respectively, and sheath, auxiliary, and sweep gas flow rates of 45, 10, and 0 (arbitrary units), respectively. The mass resolutions for the first (Q1) and third (Q3) quadrupoles were set at 0.7 and 1.2 Da, respectively, and the pressure of the collision gas (argon) in the second quadrupole (Q2) was 1.5 mTorr. The instrument control, data acquisition, and processing were performed using Xcalibur version 4.5 (ThermoFisher Scientific, San Jose, CA, USA).

Chromatographic separations were achieved by gradient elution onto a 2.1 mm × 75 mm C18 Xselect^®^ HSS T3 3.5 µm column Waters^®^ (Milford, CT, USA). The mobile phases consisted of 0.2% FA in water (A) and ACN (B); the flow rate was set to 0.3 mL/min and the injection volume to 10 µL. The optimized gradient program started with 50% B, increased to 60% B at 0.5 min, 80% B at 1.5 min, then 90% B at 3.5 min. A 1.5-min column washing step was then performed at 98% B, followed by a 2-min re-equilibration step at the initial gradient conditions (50% B).

### 3.5. Validation Procedure

The method was validated in compliance with the European Medicines Agency (EMA) ICH M10 scientific guidelines on bioanalytical method validation [23].

#### 3.5.1. Selectivity, Specificity, Crosstalk, and Carryover

The method selectivity was confirmed by injecting six blank plasma samples from drug-free subjects to ensure no interfering peaks appeared at the retention times of the target analytes. The peak area of <20% analyte of the lowest CAL or <5% of the IS at the respective retention times of the analytes are considered negligible according to EMA ICH M10 guidelines.

A key challenge arises from the potential in-source fragmentation of the LUM glucuronide metabolite, which may produce the parent drug ion and would lead to the overestimation of the LUM, in case of incidental co-elution with the parent drug. To evaluate the potential interference from the glucuronide metabolite, we assessed whether in-source dissociation during ESI could generate detectable parent drug ions at the selected mass transitions and retention time. This transition was followed solely for qualitative monitoring without quantification.

To assess crosstalk, blank plasma (CAL0) was precipitated with the IS solution, while a CAL6 sample was precipitated with the solvent of the IS only, i.e., acetonitrile (ACN). The crosstalk was quantified by calculating the ratio of the peak areas of CAL0/CAL1 for IS interference and CAL6 without IS/CAL1 for analyte interference. The acceptance criteria were set at ≤20% interference of the IS in analyte detection and ≤5% interference of the analyte in IS detection, following EMA guidelines.

Finally, the carryover was evaluated by injecting a solution containing all the analytes (IVA, LUM, TEZ, ELX, IVA-M1, TEZ-M1, ELX-M23) at the lowest (CAL1) and then at the highest concentration (CAL6) with three subsequent blank injections (blank plasma extract without IS). The signal detected in the blank sample was compared with CAL1 to determine the carryover percentages.

#### 3.5.2. Evaluation of Matrix Effect

Matrix effects were assessed both qualitatively and quantitatively. The qualitative assessment was performed by the direct infusion of analytes while simultaneously analyzing blank plasma using LC-MS/MS. This was achieved by modifying the MS inlet tubing with a “T” fitting to allow for the continuous monitoring of potential matrix-induced ion suppression or enhancement.

For the quantitative matrix effect evaluation, ten blank EDTA plasma samples from drug-free volunteers were spiked at three concentration levels (QCL2, QCM1, and QCM2). Following standard sample processing, the analyte recoveries were quantified and compared with the theoretical concentrations. The matrix effect was considered negligible and validated if the accuracy deviations remained within ±15%, as per EMA ICH M10 guidelines.

#### 3.5.3. Limit of Quantification and Linearity

The lower limit of quantification (LLOQ) was determined as the lowest analyte concentration that could be reliably quantified with an acceptable accuracy and precision, ensuring reproducible results with minimal uncertainty. The LLOQ threshold adhered to the EMA acceptability criteria of a ±20% deviation from the nominal values.

The limit of detection (LOD) was defined as the lowest analyte concentration distinguishable from background noise at a signal-to-noise (S/N) ratio of ≥3. The LOD was established experimentally through serial dilutions of CAL1 and comparison with blank plasma responses.

Linearity was evaluated by analyzing the calibration standards prepared in plasma, covering a concentration range encompassing the concentrations expected to occur in patients. Calibration curves were assessed based on regression parameters (quadratic log-log fitting), including the slope, y-intercept, and residual sum of squares (R^2^ > 0.99). A graphical assessment was performed to visualize the linearity deviations.

#### 3.5.4. Trueness and Precision

Intra- and inter-assay accuracy and precision were determined over three different days.

A series of replicate analyses (*n* = 3) were conducted on five QC validation sample levels (L1, L2, M1, M2, H) to evaluate both intra-assay (within a single analytical run) and inter-assay (across multiple runs) accuracy and precision, as part of the validation process. Among these five levels, three were selected later as QC levels for routine analysis (QC1, QC2, QC3). For this experiment, the calibrators were prepared in duplicate.

To assess the dilution integrity, QC samples with concentrations exceeding the highest calibrator (twice the highest QC level) were diluted tenfold using five different blank plasma samples, ensuring their adjusted concentrations fell within the validated plasma range.

Trueness was assessed by calculating bias, defined as the percentage difference between the measured and expected concentrations at each concentration level.

Precision was reported as relative standard deviations, and was determined through repeatability (intraday variability) and intermediate precision (variability observed within and across different days) at each concentration level. β-expectation tolerance intervals were used to define the range within which β% of the future measurements are expected to fall, providing an estimation of the total method uncertainty (MU). These intervals include both systematic and random errors [24,25,26]. Accuracy profiles, graphically representing the total analytical error, were generated using β-expectation tolerance with a β-value of 80%. For bioanalytical purposes, the acceptance limits were established at ±30% [27]. The lowest limit of quantification (LLOQ) was determined as the lowest level at which the β-expectation tolerance interval crosses the acceptance limits.

Various calibration models were assessed to determine the most suitable approach for characterizing the response-to-concentration relationship. The optimal model was selected based on estimates of trueness and precision, utilizing up to six of the eight initially tested calibrators. The decision was also influenced by the narrowest β-expectation tolerance interval and the LLOQ [28]. Finally, the MU was derived from validation phase data, setting the β-value at 0.95.

#### 3.5.5. Stability Studies

Stability experiments were conducted to assess the analyte integrity under various storage and handling conditions, including: (i) short-term stability: plasma and whole blood samples were aliquoted and stored at room temperature and at +4 °C, and then analyzed at multiple time points (0, 2, 4, 6, 24, 48, and 72 h); (ii) post-precipitation stability: extracted samples were stored in the autosampler maintained at +4 °C and re-analyzed after 24 h; (iii) freeze-thaw stability: aliquots were subjected to three freeze-thaw cycles (−80 °C) before reanalysis; and (iv) analyte stability in whole blood: due to potential ex vivo metabolism, separate experiments were conducted for the parent molecules (IVA, LUM, TEZ, ELX) and their active metabolites (IVA-M1, TEZ-M1, ELX-M23).

Stability was considered acceptable if the results remained within ±15% of the initial (*t* = 0) concentrations, per EMA recommendations.

#### 3.5.6. Inter-Laboratory Comparisons

As a proficiency program has not yet been established, inter-laboratory comparisons (ILCs) were organized with two European laboratories to evaluate the performance and accuracy of the method, as previously performed for other drug classes [29]. Since neither laboratory’s assay methods could cover all the target molecules independently, some analytes were measured in both laboratories for validation purposes, while others were analyzed exclusively in one laboratory or the other. Additionally, due to sample volume constraints, different samples were sent to each laboratory, ensuring that the same samples were analyzed in at least two laboratories. Each laboratory was blinded to the sample concentrations and analyzed the sample according to their method. Bias was determined by comparing the results obtained from the two external laboratories with those from our laboratory, with the acceptance criteria arbitrarily established at ±15%.

### 3.6. Clinical Application of the Method

Initially, a small number of patients’ blood samples were analyzed as laboratory QC analyses for the formal demonstration of assay applicability. After extensive analytical method validation, the caftors’ levels were determined in samples from patients treated with IVA + LUM dual therapy or IVA + TEZ + ELX triple therapy, collected within the framework of an observational population PK–pharmacodynamics study. All patients provided written informed consent for the donation of a 5 mL aliquot of blood (EDTA) taken at their usual medical follow-up visit, for scientific purposes. The study protocol has been approved by the Institutional Ethics Committee CER-VD (protocol 2024-01337).

Blood samples (EDTA) from patients were centrifuged at 12,700 rpm for 10 min at 4 °C, and the collected plasma was directly stored at −80 °C until the batch-wise analysis.

To preliminarily evaluate the applicability of the method to alternative biological matrices, particularly human breast milk, a calibration curve and QC were prepared in this matrix, and initial quantification tests were performed on breast milk samples from a consenting breast-feeding donor. These exploratory analyses provide preliminary insights into the method’s feasibility and its potential for future validation in lactation studies and neonatal PK assessments.

## 4. Conclusions

An LC-MS/MS method was successfully developed and validated for the simultaneous quantification of ivacaftor, lumacaftor, tezacaftor, elexacaftor, and their active metabolites in human plasma. The method complies with the ICH M10 guidelines for bioanalytical method validation, demonstrating a satisfactory performance across all key parameters, including selectivity, specificity, linearity, accuracy, precision, matrix effects, and stability.

An inter-laboratory comparison conducted with two independent European laboratories further supported the reproducibility and accuracy of the method. Nonetheless, the current absence of a formal proficiency testing program and certified reference materials for this drug class represent limitations for broader regulatory acceptance.

Preliminary clinical applications, including the analysis of patient plasma and breast milk samples, demonstrated the method’s applicability in real-world settings. However, additional validation is necessary for matrices beyond plasma (e.g., sputum, ex vivo cell extracts, etc.).

Given its low sample volume requirement and the adequacy of the analytical sensitivity with regard to the observed plasma levels, the method shows potential for use in pediatric pharmacokinetic studies and in general for therapeutic drug monitoring (TDM).

The implementation of this analytical tool in the real world constitutes the first essential step towards TDM of caftors and paves the way to characterize the association between drug exposure and the efficacy or safety of caftors in patients with cystic fibrosis, with the ultimate aim of tailoring treatment to individuals so that all patients would benefit to the maximal potential benefit of these revolutionary therapies.

## Figures and Tables

**Table 1:** Optimized MS/MS parameters of the analytes and their respective metabolites and stable isotopically labelled IS.

Compound	Precursor Ion (m/z)	Production (m/z)	CE [V]	RT [min]	Internal Standard
Ivacaftor	393.3	337.167	14	2.66	Ivacaftor-D4
Lumacaftor	453.3	413	26	2.57	Lumacaftor-D4
Tezacaftor	521.3	449.083	21	1.90	Tezacaftor-D4
Elexacaftor	598.3	422.333	26	3.36	Elexacaftor-D3
Ivacaftor-M1	409.3	353.083	16	1.66	Voriconazole-D3
Tezacaftor-M1	519.3	501.25	17	2.19	Tezacaftor-D4
Elexacaftor-M23	584.3	422.583	25	2.89	Elexacaftor-D3
Ivacaftor-D4	397.3	341.167	14	2.66	
Lumacaftor-D4	457.3	417	26	2.57	
Tezacaftor-D4	525.3	453.25	22	1.90	
Elexacaftor-D3	601.3	422.25	26	3.36	
Voriconazole-D3	353.1	284.2	15	1.40	

**Table 2:** Trueness and accuracy of the tested analytes over the validated range.

			Precision	
Compound	Concentration [µg/mL]	Trueness (%)	Repeatability (%)	Intermediate Precision (%)
Ivacaftor	0.05	100.4 ± 16	1.9	7.1
	0.1	98.0 ± 10.3	2.7	4.6
	0.4	102.2 ± 8	2.2	3.5
	2.5	102.8 ± 3.9	1.4	1.7
	5	99.5 ± 8.3	2.0	3.7
Lumacaftor	0.4	100.5 ± 4.3	1.9	1.9
	0.8	100.0 ± 14.6	6.4	6.4
	3.2	103.5 ± 4.3	1.5	1.9
	20	101.8 ± 6.9	1.8	3.1
	40	99.9 ± 7.1	3.1	3.1
Tezacaftor	0.1	99.3 ± 10	2.4	4.4
	0.2	96.7 ± 6.3	1.8	2.8
	0.8	100.3 ± 9.2	3.7	4.1
	5	102.9 ± 3	1.3	1.3
	10	100.2 ± 5.9	1.4	2.6
Elexacaftor	0.15	102.3 ± 8.7	1.1	3.8
	0.3	101.2 ± 8.2	3.6	3.6
	1.2	101.4 ± 5.8	2.5	2.5
	7.5	102.9 ± 6.7	2.8	3.0
	15	99.1 ± 9.7	4.3	4.3
Ivacaftor-M1	0.05	98.0 ± 19.3	6.7	8.5
	0.1	97.7 ± 18.2	8.0	8.0
	0.4	98.2 ± 15	6.6	6.6
	2.5	103.3 ± 10.9	2.8	4.8
	5	101.1 ± 6.8	2.0	3.0
Tezacaftor-M1	0.15	107.8 ± 15.4	3.6	6.8
	0.3	107.9 ± 18.3	8.1	8.1
	1.2	104.9 ± 7.8	3.4	3.4
	7.5	104.2 ± 5.1	1.6	2.2
	15	100.8 ± 10.2	2.9	4.5
Elexacaftor-M23	0.15	89.8 ± 24.6	4.0	10.9
	0.3	91.0 ± 14.5	4.9	6.4
	1.2	102.2 ± 13.8	4.9	6.1
	7.5	107.3 ± 15.2	5.6	6.7
	15	99.2 ± 13.3	4.0	5.9

**Table 3:** Final concentrations (µg/mL) of calibration solutions (CALs) and quality controls (QCs) reported for the different analytes in plasma.

Molecule	CAL 1	CAL 2	CAL 3	CAL 4	CAL 5	CAL 6	QC 1	QC 2	QC 3
Ivacaftor	0.05	0.10	0.20	0.50	1.25	5.00	0.15	0.75	3.75
Lumacaftor	0.40	0.80	1.60	4.00	10.00	40.00	1.20	6.00	30.00
Tezacaftor	0.10	0.20	0.40	1.00	2.50	10.00	0.30	1.50	7.50
Elexacaftor	0.15	0.30	0.60	3.00	7.50	15.00	0.45	2.25	11.25
Ivacaftor-M1	0.05	0.10	0.20	1.00	2.50	5.00	0.15	0.75	3.75
Tezacaftor-M1	0.15	0.30	0.60	3.00	7.50	15.00	0.45	2.25	11.25
Elexacaftor-M23	0.15	0.30	0.60	3.00	7.50	15.00	0.45	2.25	11.25

## Data Availability

Data are contained within the article or Supplementary Materials.

## Supplementary Materials

The following supporting information can be downloaded at: https://www.mdpi.com/article/10.3390/molecules30091866/s1, Figure S1: Optimization of mobile phase composition, Figure S2: Optimization of MS parameters, Figure S3: Optimization of the extraction procedure, Table S1: Method selectivity, Table S2: Method linearity, Table S3: Evaluation of the matrix effect, Table S4: Stability of the extracted analytes, Table S5: Evaluation of the dilution integrity, Figure S4: Identification of LUM glucuronide, Figure S5: Caftors quantification in human breast milk, Figure S6: Assay interferences with common CF co-medications.
