# Supplementary material for: A Liquid Chromatography-Tandem Mass Spectrometry Method for the Quantification of Cystic Fibrosis Drugs (Caftors) in Plasma and Its Application for Therapeutic Monitoring
Source: Molecules. 2025 Apr 22;30(9):1866. doi: 10.3390/molecules30091866 (PMC12073025; doi:10.3390/molecules30091866)

Supporting Information

Development of a Liquid Chromatography-Tandem Mass Spectrometry Method for the Quantification of Cystic Fibrosis Drugs (Caftors) in Plasma and Its Application for Therapeutic Monitoring

Valentina D'Atri[1*], Fabrizio Corrado[1*], François Versace[1], Susana Alves[1], Thomas Mercier[1], Monia Guidi[1,2], Paul Thoueille[1], Sylvain Blanchon[3], Angela Koutsokera[4], Michael Vogeser[5], Catia Marzolini[1], François Girardin[1], Georgia Mitropoulou[4], Zisis Balmpouzis[4], Isabelle Rochat[3], Alain Sauty[4,6], Laurent A. Decosterd[1], Eva Choong[1**]

[1]Service and Laboratory of Clinical Pharmacology, Department of Laboratory Medicine and Pathology, Lausanne University Hospital and University of Lausanne, Lausanne, Switzerland

[2]Service of Pneumology, Adult Cystic Fibrosis Unit, Pourtalès Hospital, Neuchâtel, Switzerland.

[3]Division of Pulmonology, Unit of Adult Cystic Fibrosis Unit and CFTR-related disorders, Lausanne University Hospital and University of Lausanne, Lausanne, Switzerland

[4]Service of Pediatrics, Pediatric Pneumology and Cystic Fibrosis Unit, Lausanne University Hospital and University of Lausanne, Lausanne, Switzerland

TABLE OF CONTENTS

**Figure S1**	Optimization of mobile phase composition……………………….……………	S1
**Figure S2**	Optimization of MS parameters…………………………………….............	S2
**Figure S3**	Optimization of extraction procedure…………………………………..	S2
**Table S1**	Method selectivity……………………………………………………………..	S3
**Table S2**	Method linearity…………………………………………………................	S4
**Table S3**	Evaluation of matrix effect…………………………………………………….	S5
**Table S4**	Stability of the extracted analytes……………………………...................	S8
**Table S5**	Evaluation of dilution integrity………………………………………............	S9
**Figure S4**	Identification of lumacaftor glucuronide…..……………………………	S12
**Figure S5**	Caftors quantification in human breast milk………………...…………	S13
**Figure S6**	Assay interferences with common CF co-medications……..………	S14-S17

Figure S1. Mobile phase optimization. Chromatographic profiles of ivacaftor (IVA), ivacaftor-M1 (IVA-M1), lumacaftor (LUM), tezacaftor (TEZ), tezacaftor-M1 (TEZ-M1), elexacaftor (ELX), and elexacaftor-M23 (ELX-M23), at a concentration of 100 ng/mL. The chromatograms were obtained using two different mobile phases: H_2O MilliQ + 0.1 % AF *vs* H_2O MilliQ + 0.2 % AF. Corresponding internal standards are not shown.

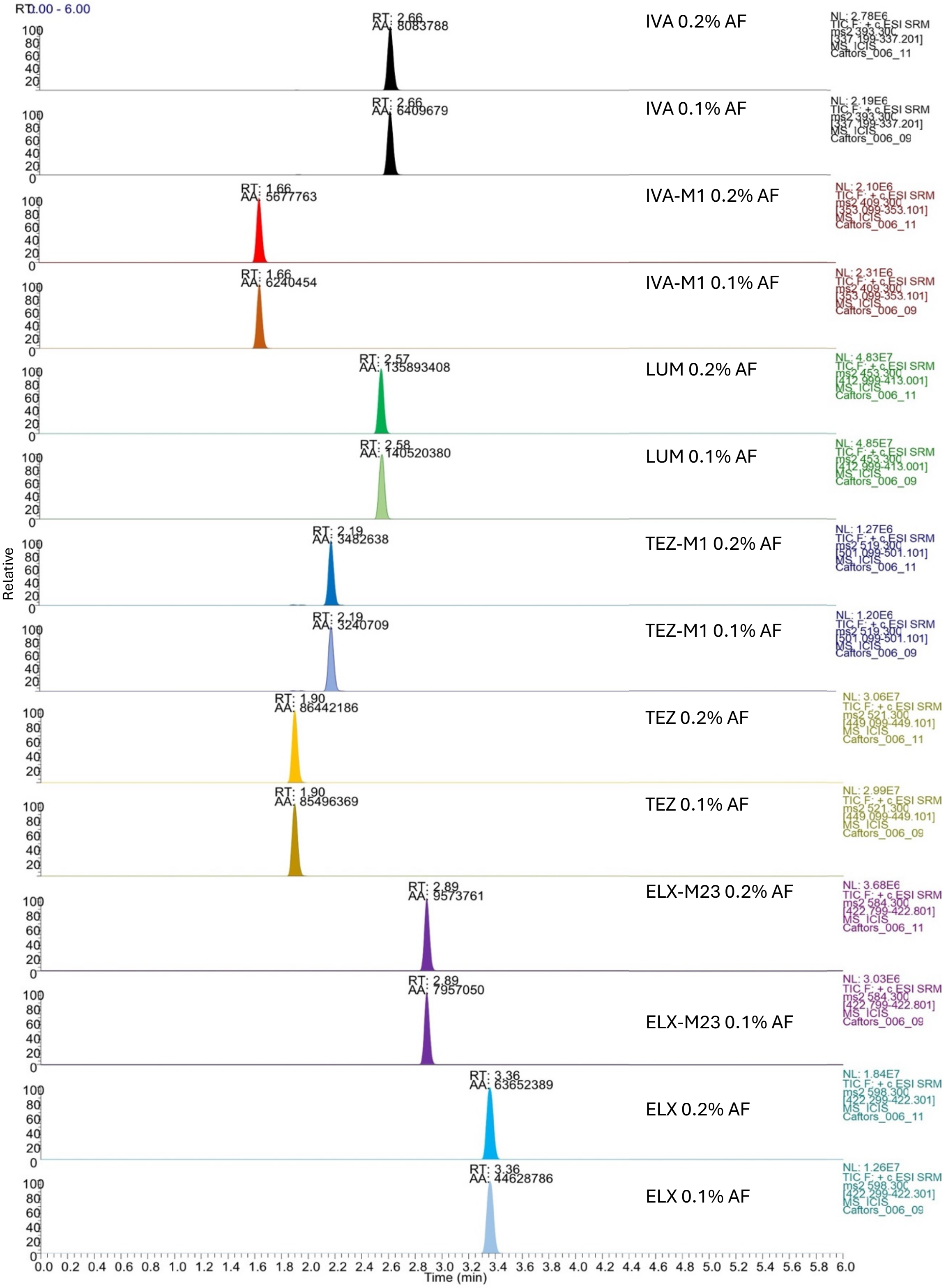

Figure S2. MS parameters optimization. Impact of vaporization temperature on analyte peak area.

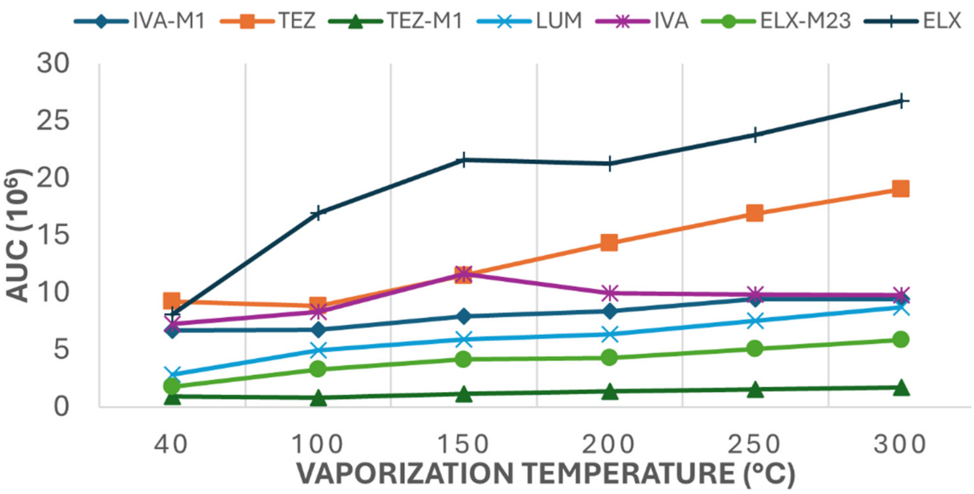

Figure S3. Sample extraction optimization. Impact of the extraction solvent on analyte peak area. Precipitation step performed in triplicate by mixing 50µL plasma with 150µL ACN or 150µL MeOH. Average areas are reported.

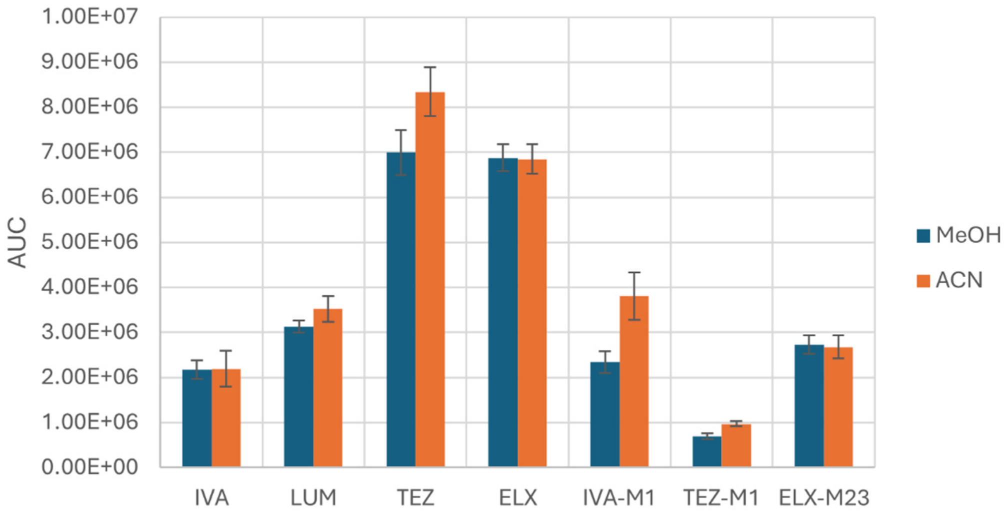

Table S1. Method selectivity. Interference of analytes at the LLOQ (CAL1) and internal standards measured in 6 blank plasma.

	IVA		LUM		TEZ		ELX	
	Interf. analyte	Interf. IS	Interf. analyte	Interf. IS	Interf. analyte	Interf. IS	Interf. analyte	Interf. IS
**Matrix 1**	0.02%	0.00%	0.04%	0.00%	0.02%	0.00%	0.09%	0.00%
**Matrix 2**	0.14%	0.00%	0.06%	0.00%	0.00%	0.00%	0.05%	0.00%
**Matrix 3**	0.00%	0.00%	0.02%	0.00%	0.00%	0.00%	0.01%	0.00%
**Matrix 4**	0.03%	0.00%	0.03%	0.00%	0.03%	0.00%	0.00%	0.00%
**Matrix 5**	0.00%	0.00%	0.02%	0.00%	0.00%	0.00%	0.00%	0.00%
**Matrix 6**	0.03%	0.00%	0.02%	0.00%	0.00%	0.00%	0.02%	0.00%

	IVA-M1		TEZ-M1		ELX-M23	
	Interf. analyte	Interf. IS	Interf. analyte	Interf. IS	Interf. analyte	Interf. IS
**Matrix 1**	0.00%	0.02%	0.63%	0.00%	0.02%	0.00%
**Matrix 2**	0.04%	0.03%	0.36%	0.00%	0.00%	0.00%
**Matrix 3**	0.16%	0.02%	0.72%	0.00%	0.00%	0.00%
**Matrix 4**	0.01%	0.02%	1.22%	0.00%	0.02%	0.00%
**Matrix 5**	0.00%	0.03%	0.51%	0.00%	0.00%	0.00%
**Matrix 6**	0.00%	0.02%	0.94%	0.00%	0.00%	0.00%

Table S2. Method linearity. Linear trends for 3 sets of calibration curve measurements for each analyte.

Ivacaftor :

	Day 1	Day 2	Day 3
**Slope**	**0.96327**	**1.01862**	**1.01358**
**y-intercept**	**0.02754**	**-0.00059**	**0.01220**
**R^2**	**0.99872**	**0.99934**	**0.99941**

Lumacaftor :

	Day 1	Day 2	Day 3
**Slope**	**0.98410**	**1.00968**	**1.00563**
**y-intercept**	**0.19366**	**0.09147**	**-0.03505**
**R^2**	**0.99874**	**0.99781**	**0.99951**

Tezacaftor :

	Day 1	Day 2	Day 3
**Slope**	**0.98389**	**1.02527**	**1.00664**
**y-intercept**	**0.03499**	**-0.00407**	**0.02657**
**R^2**	**0.99926**	**0.99978**	**0.99937**

Elexacaftor :

	Day 1	Day 2	Day 3
**Slope**	**0.97363**	**0.99921**	**1.00873**
**y-intercept**	**0.09404**	**0.01693**	**0.04859**
**R^2**	**0.99826**	**0.99950**	**0.99903**

Ivacaftor-M1 :

	Day 1	Day 2	Day 3
**Slope**	**0.99514**	**1.01473**	**1.03456**
**y-intercept**	**0.01598**	**0.01732**	**-0.02841**
**R^2**	**0.99906**	**0.99881**	**0.99817**

Tezacaftor-M1 :

	Day 1	Day 2	Day 3
**Slope**	**1.00286**	**0.98173**	**1.04717**
**y-intercept**	**0.08074**	**0.09655**	**-0.00979**
**R^2**	**0.99841**	**0.99789**	**0.9916**

Elexacaftor-M23 :

	Day 1	Day 2	Day 3
**Slope**	**0,94520**	**1,02540**	**1,03471**
**y-intercept**	**0,07844**	**0,06275**	**0,07828**
**R^2**	**0,99752**	**0,99287**	**0,99825**

Table S3. Evaluation of matrix effect. The matrix effect for each analyte was evaluated by analyzing 3 QCs, each prepared using matrix from 10 different sources (8 EDTA- and 2 hemolysed-matrix samples).

a) Ivacaftor

Matrix	Accuracy in reference to nominal concentration		
	0,1 µg/mL	0,4 µg/mL	2,5 µg/mL
**EDTA-1**	112%	109%	109%
**EDTA-2 Hemo**	106%	115%	111%
**EDTA-3**	113%	106%	106%
**EDTA-4**	110%	109%	112%
**EDTA-5**	103%	104%	111%
**EDTA-6**	113%	111%	109%
**EDTA-7**	110%	114%	112%
**EDTA-8**	113%	102%	110%
**EDTA-9**	114%	105%	107%
**EDTA-10 Hemo**	111%	111%	105%
**Average**	**110%**	**109%**	**109%**
**CV**	**3%**	**4%**	**2%**

b) Lumacaftor

Matrice	Accuracy in reference to nominal concentration		
	0,1 µg/mL	0,4 µg/mL	2,5 µg/mL
**EDTA-1**	112%	109%	109%
**EDTA-2 Hemo**	106%	115%	111%
**EDTA-3**	113%	106%	106%
**EDTA-4**	110%	109%	112%
**EDTA-5**	103%	104%	111%
**EDTA-6**	113%	111%	109%
**EDTA-7**	110%	114%	112%
**EDTA-8**	113%	102%	110%
**EDTA-9**	114%	105%	107%
**EDTA-10 Hemo**	111%	111%	105%
**Average**	**110%**	**109%**	**109%**
**CV**	**3%**	**4%**	**2%**

c) Tezacaftor

Matrice	Accuracy in reference to nominal concentration		
	0,2 µg/mL	0,8 µg/mL	5 µg/mL
**EDTA-1**	109%	109%	107%
**EDTA-2 Hemo**	110%	109%	112%
**EDTA-3**	108%	110%	106%
**EDTA-4**	108%	111%	111%
**EDTA-5**	108%	107%	113%
**EDTA-6**	107%	113%	110%
**EDTA-7**	113%	111%	112%
**EDTA-8**	107%	109%	107%
**EDTA-9**	111%	110%	112%
**EDTA-10 Hemo**	107%	106%	107%
**Average**	**109%**	**110%**	**110%**
**CV**	**2%**	**2%**	**2%**

d) Elexacaftor

Matrice	Accuracy in reference to nominal concentration		
	0,3 µg/mL	1,2 µg/mL	7,5 µg/mL
**EDTA-1**	110%	100%	104%
**EDTA-2 Hemo**	108%	105%	111%
**EDTA-3**	111%	95%	104%
**EDTA-4**	110%	102%	108%
**EDTA-5**	105%	101%	111%
**EDTA-6**	111%	107%	110%
**EDTA-7**	112%	113%	111%
**EDTA-8**	110%	86%	108%
**EDTA-9**	109%	96%	107%
**EDTA-10 Hemo**	109%	98%	106%
**Average**	**109%**	**100%**	**108%**
**CV**	**2%**	**7%**	**2%**

e) Ivacaftor-M1

Matrice	Accuracy in reference to nominal concentration		
	0,11 µg/mL	0,44 µg/mL	2,75 µg/mL
**EDTA-1**	98%	98%	93%
**EDTA-2 Hemo**	103%	102%	102%
**EDTA-3**	107%	110%	103%
**EDTA-4**	106%	100%	94%
**EDTA-5**	90%	97%	102%
**EDTA-6**	104%	108%	98%
**EDTA-7**	105%	99%	100%
**EDTA-8**	107%	98%	100%
**EDTA-9**	105%	114%	102%
**EDTA-10 Hemo**	94%	99%	100%
**Average**	**102%**	**103%**	**99%**
**CV**	**6%**	**6%**	**4%**

f) Tezacaftor-M1

Matrice	Accuracy in reference to nominal concentration		
	0,39 µg/mL	1,56 µg/mL	9,75 µg/mL
**EDTA-1**	97%	93%	91%
**EDTA-2 Hemo**	95%	93%	93%
**EDTA-3**	103%	99%	90%
**EDTA-4**	95%	104%	92%
**EDTA-5**	105%	93%	98%
**EDTA-6**	97%	106%	94%
**EDTA-7**	102%	97%	94%
**EDTA-8**	96%	95%	93%
**EDTA-9**	108%	96%	101%
**EDTA-10 Hemo**	95%	97%	90%
**Average**	**99%**	**97%**	**94%**
**CV**	**5%**	**5%**	**4%**

g) Elexacaftor-M23

Matrice	Accuracy in reference to nominal concentration		
	0,36 µg/mL	1,44 µg/mL	11,625 µg/mL
**EDTA-1**	95%	95%	**83%**
**EDTA-2 Hemo**	93%	97%	92%
**EDTA-3**	103%	97%	91%
**EDTA-4**	101%	106%	99%
**EDTA-5**	101%	108%	96%
**EDTA-6**	106%	101%	99%
**EDTA-7**	99%	108%	105%
**EDTA-8**	96%	91%	104%
**EDTA-9**	102%	105%	**115%**
**EDTA-10 Hemo**	103%	**127%**	113%
**Average**	**100%**	**104%**	**100%**
**CV**	**4%**	**10%**	**10%**

Table S4. Stability of caftors in extracted plasma evaluated over a storage period of 24 hours at 5°C (the temperature of the autosampler). A sequence containing a calibration curve in duplicate, and 3 QC in triplicate was analyzed at t0 and t24h. The area ratios obtained after 24h for each CAL and QC were compared to the area ratios obtained at t0. The percentages obtained relative to t0 (=100%) are presented.

	Ivacaftor	Lumacaftor	Tezacaftor	Elexacaftor	Ivacaftor-M1	Tezacaftor-M1	Elexacaftor-M23
CAL 1	93.26	99.89	97.78	98.20	87.41	107.06	95.26
CAL 2	94.80	98.90	96.85	97.42	84.74	109.23	95.30
CAL 3	95.04	98.82	97.70	97.09	84.34	108.70	94.60
CAL 4	94.74	101.27	96.49	97.93	87.31	101.56	89.65
CAL 5	95.02	103.86	98.68	98.96	82.88	105.74	90.59
CAL 6	94.69	106.72	98.20	96.50	83.02	105.61	92.19
QC 1	93.63	99.54	96.65	97.90	83.34	112.79	90.40
QC 2	93.34	101.02	97.79	97.21	83.62	112.98	93.88
QC 3	94.71	105.30	97.37	97.58	84.90	105.17	92.75

Table S5. Evaluation of dilution integrity. A dilution QC was prepared with analyte concentrations in the matrix that were 2 times greater than the ULOQ. The dilution QC was then diluted 10x with a blank matrix. Comparisons of measured concentrations with theoretical concentrations are reported.

a) Ivacaftor

Sample	Dilution factor	Theoretical concentration [µg/mL]	Measured concentration [µg/mL]	Bias
**Dil. plasma 1**	10	10	11	**13.0%**
**Dil. plasma 2**	10	10	10	**5.0%**
**Dil. plasma 3**	10	10	11	**5.5%**
**Dil. plasma 4**	10	10	11	**6.0%**
**Dil. plasma 5**	10	10	11	**6.0%**
**Dil. H2O**	10	10	10	**3.7%**

b) Lumacaftor

Sample	Dilution factor	Theoretical concentration [µg/mL]	Measured concentration [µg/mL]	Bias
**Dil. plasma 1**	10	80	90	**12.4%**
**Dil. plasma 2**	10	80	81	**0.7%**
**Dil. plasma 3**	10	80	87	**8.3%**
**Dil. plasma 4**	10	80	84	**5.3%**
**Dil. plasma 5**	10	80	88	**10.1%**
**Dil. H2O**	10	80	86	**7.8%**

c) Tezacaftor

Sample	Dilution factor	Theoretical concentration [µg/mL]	Measured concentration [µg/mL]	Bias
**Dil. plasma 1**	10	20	22.2	**11.1%**
**Dil. plasma 2**	10	20	20.9	**4.7%**
**Dil. plasma 3**	10	20	21.6	**8.2%**
**Dil. plasma 4**	10	20	21.3	**6.4%**
**Dil. plasma 5**	10	20	21.6	**7.9%**
**Dil. H2O**	10	20	20.7	**3.4%**

d) Elexacaftor

Sample	Dilution factor	Theoretical concentration [µg/mL]	Measured concentration [µg/mL]	Bias
**Dil. plasma 1**	10	30	32.8	**9.2%**
**Dil. plasma 2**	10	30	32.8	**9.2%**
**Dil. plasma 3**	10	30	32.3	**7.7%**
**Dil. plasma 4**	10	30	32.6	**8.6%**
**Dil. plasma 5**	10	30	32.1	**7.0%**
**Dil. H2O**	10	30	28.1	**-6.2%**

e) Ivacaftor-M1

Sample	Dilution factor	Theoretical concentration [µg/mL]	Measured concentration [µg/mL]	Bias
**Dil. plasma 1**	10	11	12.3	**11.7%**
**Dil. plasma 2**	10	11	11.8	**7.4%**
**Dil. plasma 3**	10	11	11.6	**5.1%**
**Dil. plasma 4**	10	11	11.2	**1.9%**
**Dil. plasma 5**	10	11	12.0	**8.9%**
**Dil. H2O**	10	11	4.3	**-60.6%**

f) Tezacaftor-M1

Sample	Dilution factor	Theoretical concentration [µg/mL]	Measured concentration [µg/mL]	Bias
**Dil. plasma 1**	10	36	38.8	**7.7%**
**Dil. plasma 2**	10	36	32.5	**-9.7%**
**Dil. plasma 3**	10	36	34.6	**-3.9%**
**Dil. plasma 4**	10	36	32.6	**-9.4%**
**Dil. plasma 5**	10	36	38.3	**6.5%**
**Dil. H2O**	10	36	29.7	**-17.6%**

g) Elexacaftor-M23

Sample	Dilution factor	Theoretical concentration [µg/mL]	Measured concentration [µg/mL]	Bias
**Dil. plasma 1**	10	30	30.2	**0.6%**
**Dil. plasma 2**	10	30	32.0	**6.7%**
**Dil. plasma 3**	10	30	33.6	**12.0%**
**Dil. plasma 4**	10	30	34.0	**13.3%**
**Dil. plasma 5**	10	30	30.4	**1.5%**
**Dil. H2O**	10	30	31.9	**6.2%**

Figure S4: Identification of lumacaftor glucuronide.

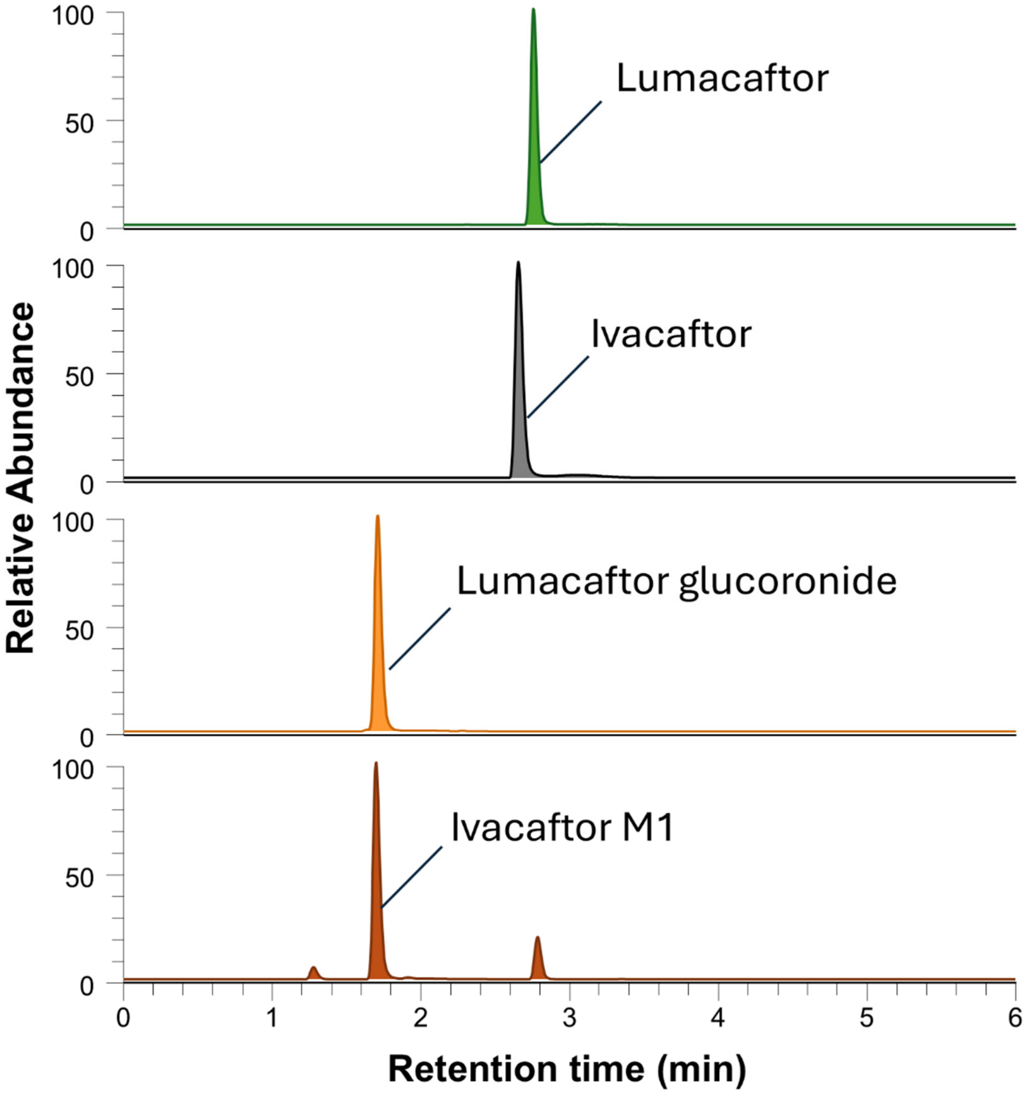

Figure S5. Chromatogram of a patient receiving Trikafta® (IVA + TEZ + ELX tri-therapy), collected 3h after dosing. Quantification performed in human breast milk. The breast milk concentrations of ivacaftor, ivacaftor-M1, elexacaftor, elexacaftor-M23, tezacaftor, and tezacaftor-M1, were 0.10, 0.38, 0.37, 0.14, 0.25, and 0.35 µg/mL, respectively. Corresponding internal standards are not shown.

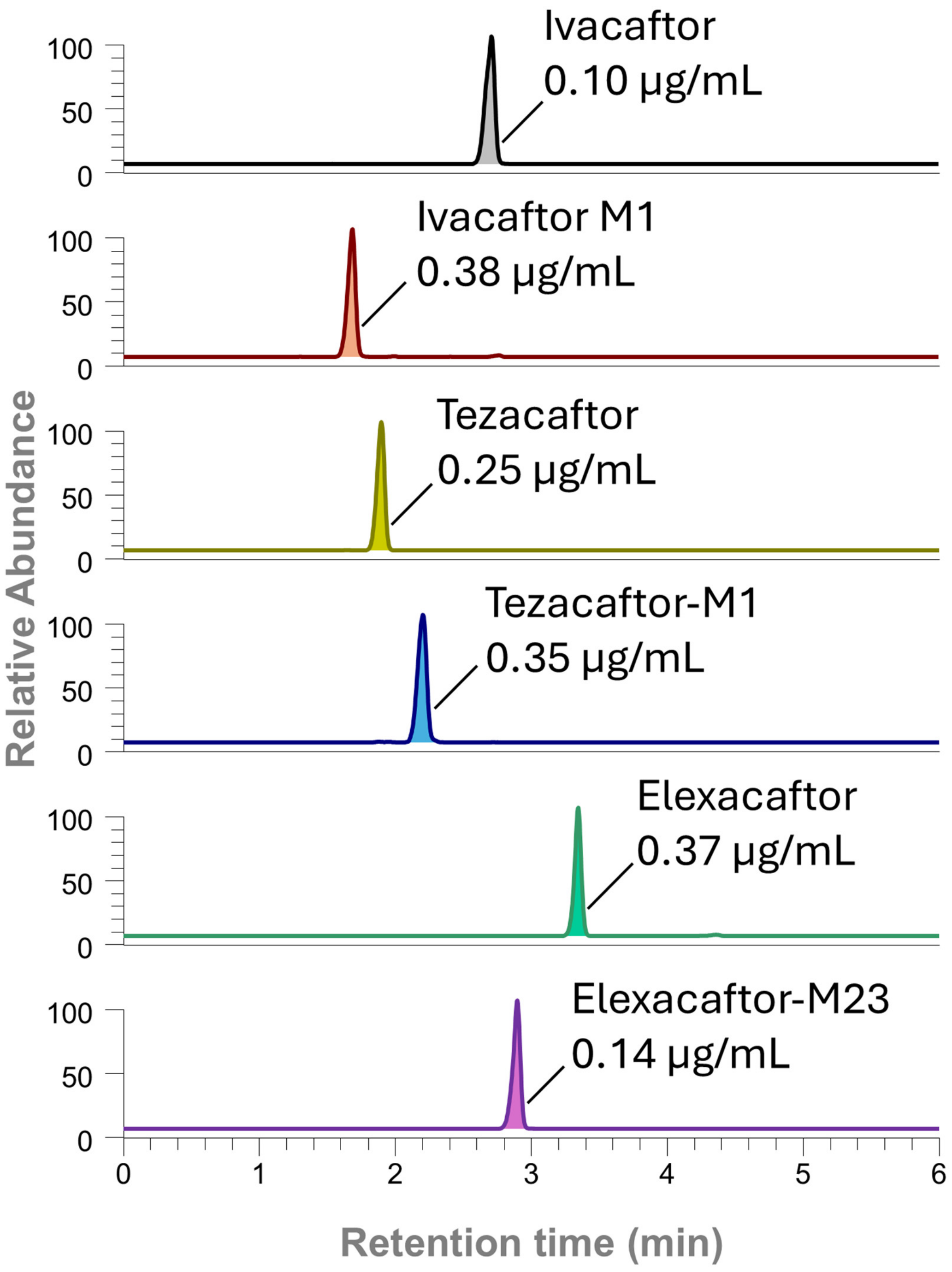

Figure S6. Assay interferences with common CF co-medications. Chromatographic profile of a caftors QC3 plasma quality control sample (IVA 3.75 µg/mL, LUM 30 µg/mL, TEZ 7.50 µg/mL, ELX 11.25 µg/mL, IVA-M1 3.75 µg/mL, TEZ-M1 11.25 µg/mL, ELX-M23 11.25 µg/mL) in comparison to known, high concentration of the potential interfering substances relevant to the patient population, namely ibuprofen 2 mg/mL, cortisone 500 ng/mL, colistin 10 mg/L, antibiotics pool 1 (imipenem 100 mg/L, cefepime 150 mg/L, meropenem 100 mg/L, cefazolin 150 mg/L, piperacillin 200 mg/L), antibiotics pool 2 (amoxicillin 100 mg/L, ceftazidime 100 mg/L, ceftriaxone 200 mg/L, daptomycin 100 mg/L, ertapenem 100 mg/L, flucloxacillin 100 mg/L, and rifampicin 10 mg/L), and antifungal drugs (fluconazole 17.9 µg/mL, voriconazole 7.74 µg/mL, posaconazole 4.96 µg/mL, OH-itraconazole 2.48 µg/mL, isavuconazole 20.4 µg/mL, and itraconazole 1.87 µg/mL). For sake of readability, the corresponding internal standards are not shown.

Caftors QC3 plasma quality control sample

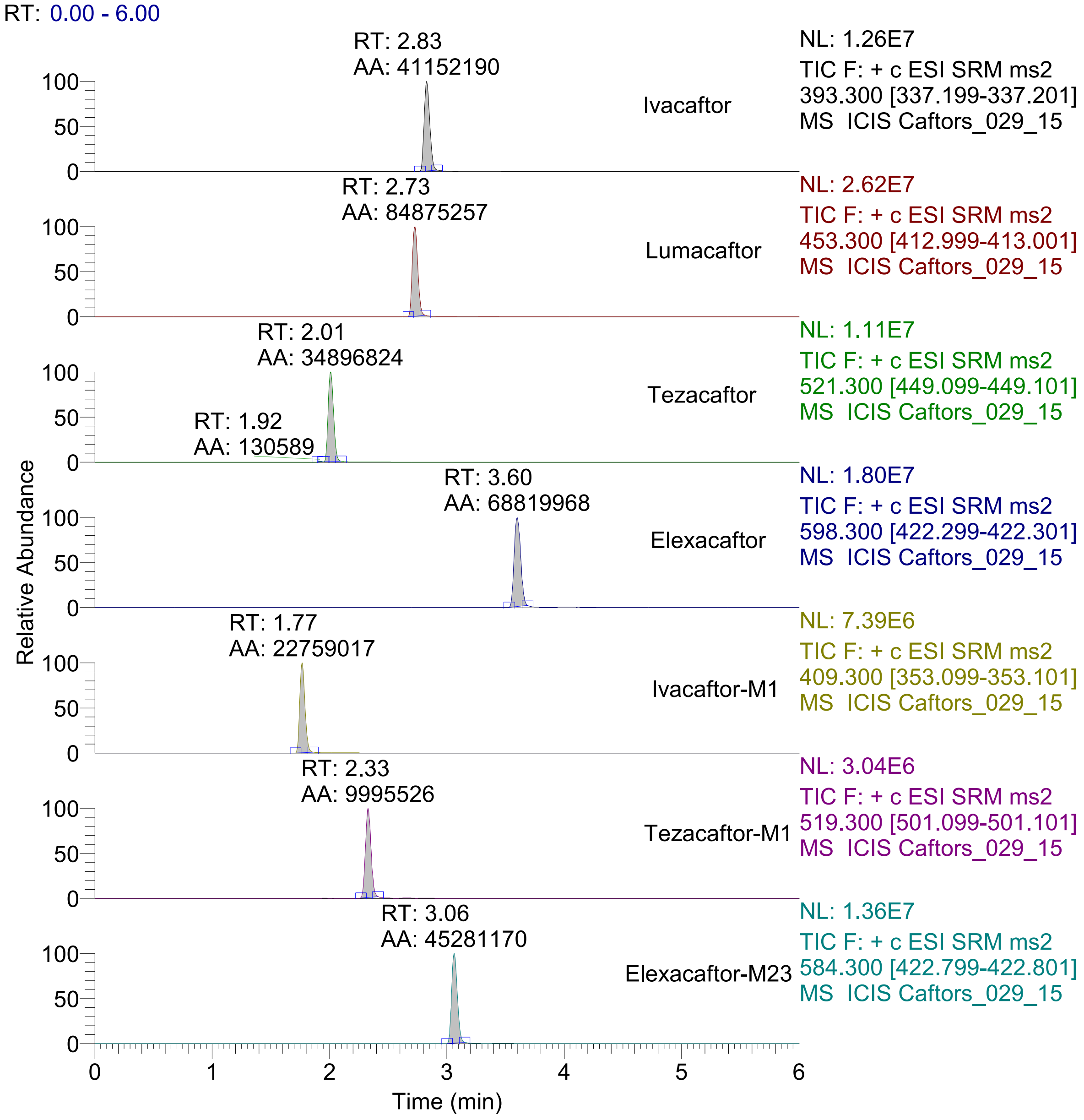

Ibuprofen 2 mg/L

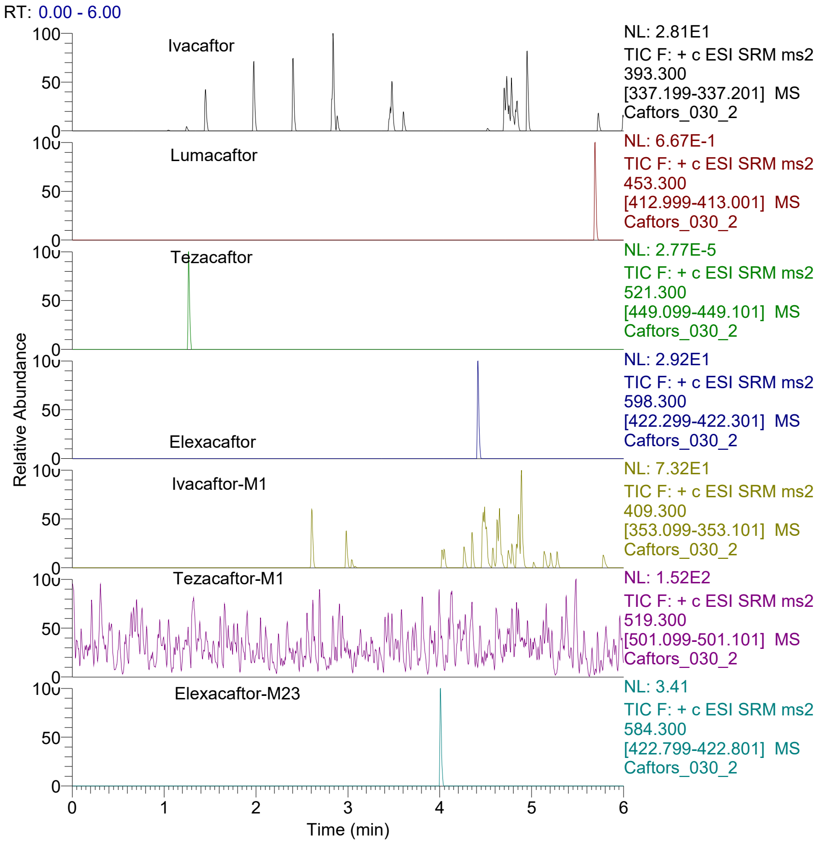

Cortisone 500 ng/mL

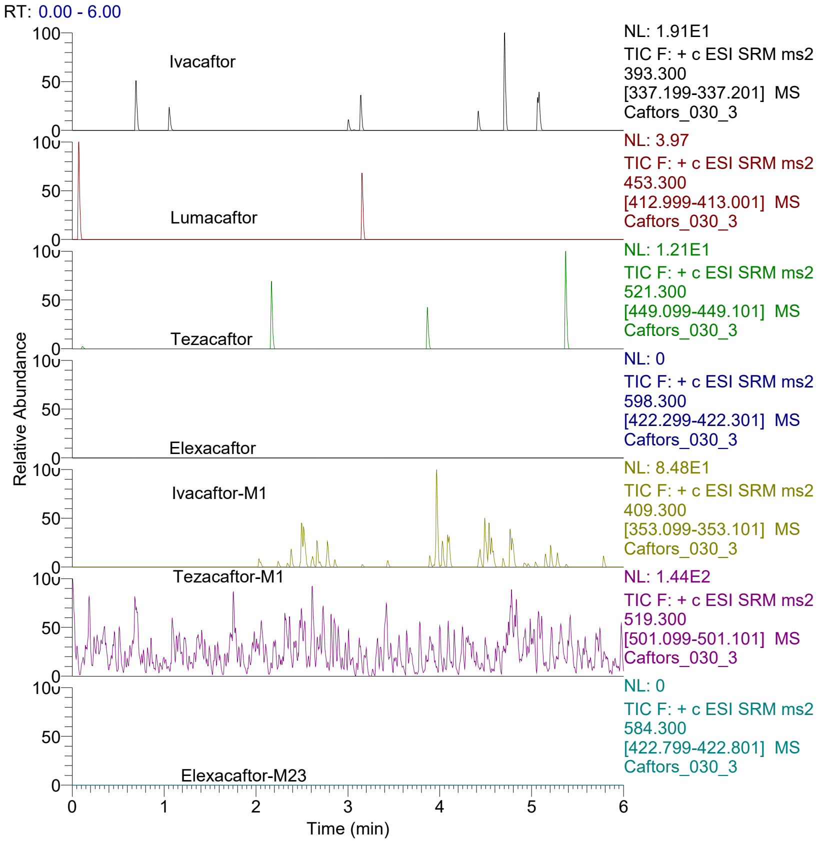

Colistin 10 mg/L

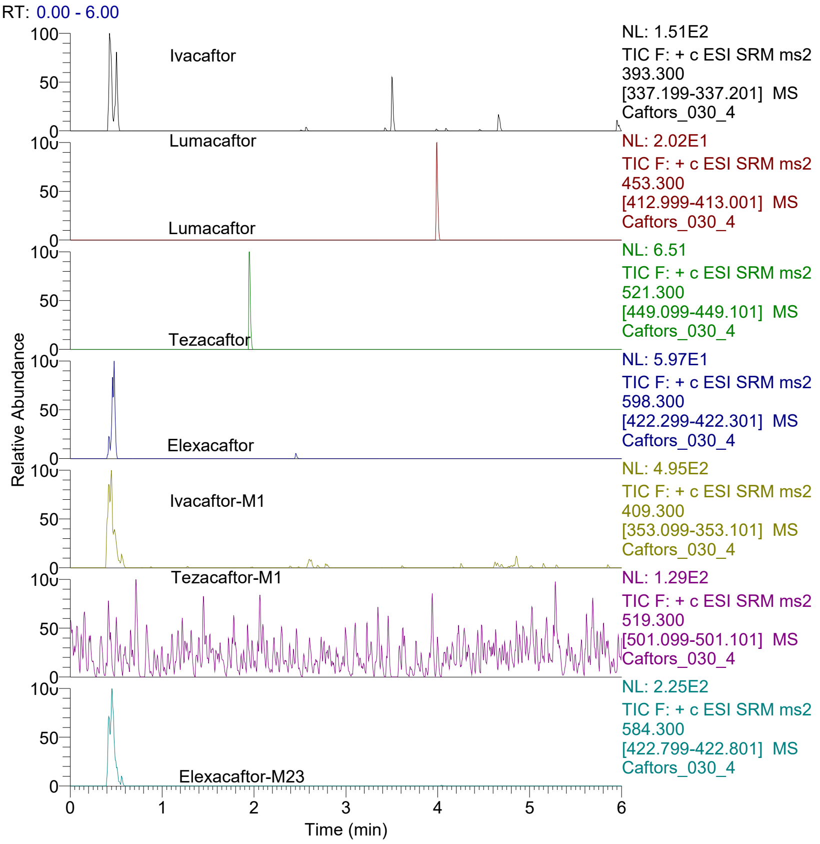

Antibiotics pool1: imipenem 100 mg/L, cefepime 150 mg/L, meropenem 100 mg/L, cefazolin 150 mg/L, piperacillin 200 mg/L.

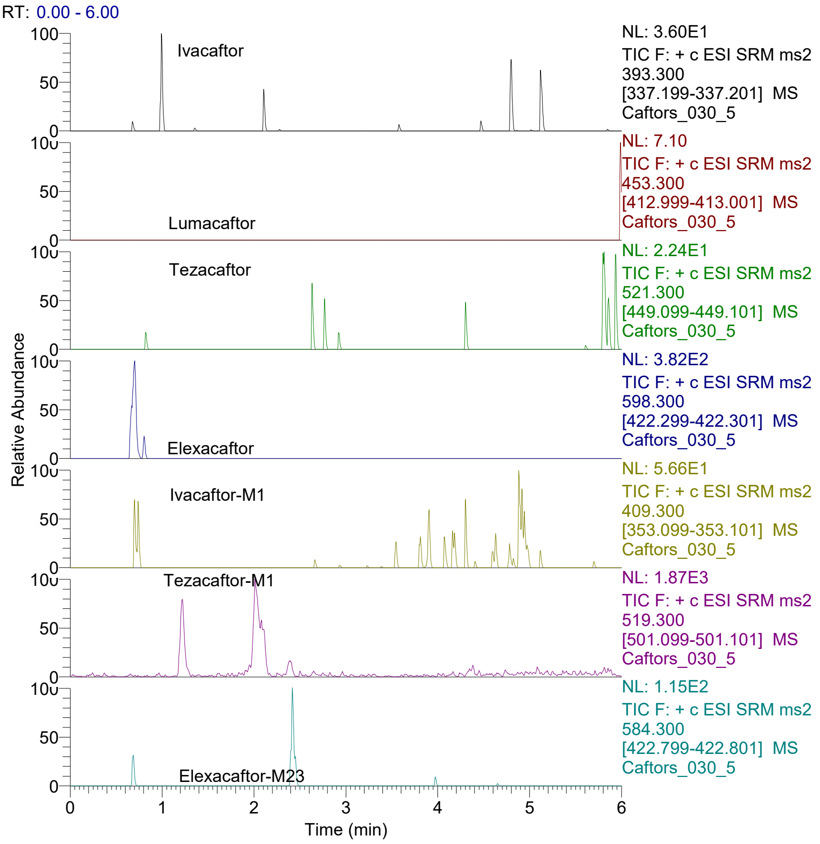

Antibiotics pool2: amoxicillin 100 mg/L, ceftazidime 100 mg/L, ceftriaxone 200 mg/L, daptomycin 100 mg/L, ertapenem 100 mg/L, flucloxacillin 100 mg/L, and rifampicin 10 mg/L.

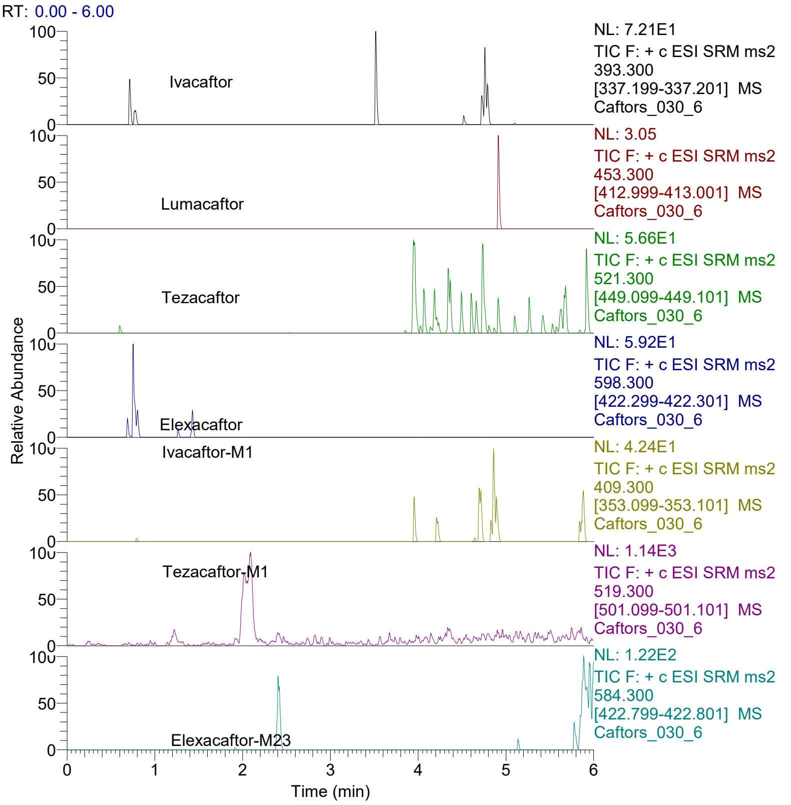

Antifungal drugs: fluconazole 17.9 μg/mL, voriconazole 7.74 μg/mL, posaconazole 4.96 μg/mL, OH-itraconazole 2.48 μg/mL, isavuconazole 20.4 μg/mL, and itraconazole 1.87 μg/mL

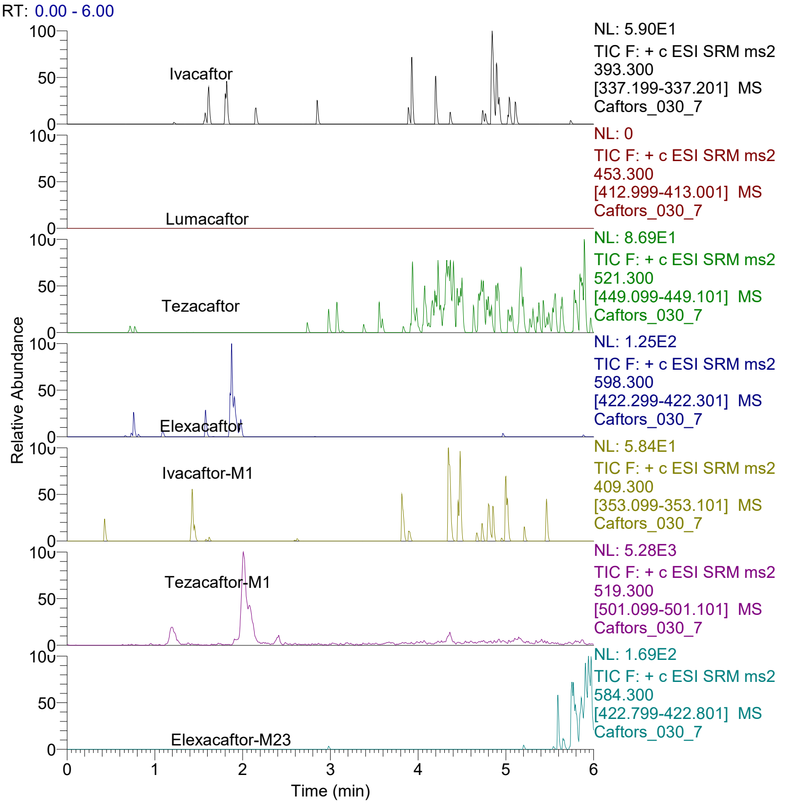

Supplement: Supplementary file 1 [file molecules-30-01866-s001.zip › molecules-3538689-supplementary.pdf]
